# A long-distance signaling loop promotes soybean nodulation and productivity

**DOI:** 10.1073/pnas.2609325123

**Published:** 2026-06-08

**Authors:** Jingbo Duan, Jinbin Wang, Runze Guo, Chancelor B. Clark, Zhuojun Luo, Xiaochong Li, Leonie Trabert, Xing-Qi Huang, W. Andy Tao, Natalia Dudareva, Gary Stacey, Blake C. Meyers, Jianxin Ma

**Affiliations:** ^a^ Department of Agronomy, Purdue University, West Lafayette, IN 47907; ^b^ Center for Plant Biology, Purdue University, West Lafayette, IN 47907; ^c^ Department of Biochemistry, Purdue University, West Lafayette, IN 47907; ^d^ Division of Plant Science & Technology, University of Missouri, Columbia, MO 65211; ^e^ Genome Center and Department of Plant Sciences, University of California, Davis, CA 95616

**Keywords:** long-distance signaling, mobile microRNA, mobile peptide, nodulation, soybean

## Abstract

Legume nodulation is initiated when soil bacteria rhizobia infect root hairs and is tightly regulated by host-derived mechanisms that restrict nodule numbers to balance the benefits of symbiotic nitrogen fixation with the plant’s growth and metabolic demands. However, how plants actively promote the initiation of nodulation to counterbalance these restrictive mechanisms and maintain an optimal level of nodulation remains largely unknown. Here, we report a systemic regulatory mechanism through which soybean (*Glycine max*) promotes rhizobial infection. We show that inoculation of soybean roots with rhizobia suppresses the biogenesis of microRNA miR4416-5p in shoots, a mobile microRNA that is transported from shoots to roots. The resulting reduction of miR4416-5p levels in roots enhances the expression of a vegetative lectin gene *Lectin 3* (*GmLe3*), which promotes rhizobial infection, thereby enhancing nodule formation and improving plant productivity under low nitrogen conditions. We further demonstrate that suppression of miR4416-5p biogenesis in shoots is triggered by the root-derived C-TERMINALLY ENCODED PEPTIDE 7 (GmCEP7), establishing a long-distance GmCEP7-miR4416-5p-*GmLe3* regulatory loop that is critical for desirable symbiotic synergy and plant productivity. Comparative genomic analysis reveals that this miR4416-5p-mediated regulatory module is absent in the model legumes *Medicago truncatula* and *Lotus japonicus* but appears to be conserved in economically important legume crops common bean (*Phaseolus vulgaris*) and pigeonpea (*Cajanus cajan*), suggesting an evolutionary innovation in nodulation control. These findings uncover a systemic mechanism that promotes rhizobial infection and highlight an evolutionary innovation in regulation of nodulation with potential implications for improving legume crop productivity under nitrogen-limited conditions.

## Significance

Legumes rely on symbiotic nitrogen fixation to sustain productivity in both agricultural and natural ecosystems, yet the mechanisms that promote the formation of nitrogen-fixing root nodules remain poorly understood. This study uncovers a systemic signaling pathway that links a root-derived hormone peptide with a shoot-derived microRNA to regulate expression of a lectin gene in soybean root hairs, thereby modulating nodulation. Manipulating this pathway by silencing the microRNA, overexpressing its target gene, or increasing expression of the peptide precursor enhances nodulation and improves plant productivity under nutrient-limited conditions. Our findings also demonstrate the value of integrating insights from both model legumes and crop species to better understand regulatory mechanisms underlying nodulation and their evolutionary innovation, while advancing strategies for sustainable agriculture.

Symbiotic nitrogen fixation (SNF) by rhizobia within the root nodules of legumes, such as the model species *Lotus japonicus* and *Medicago truncatula*, and the economically important crops soybean (*Glycine max*) and common bean (*Phaseolus vulgaris*), is a vital biological process in natural and agricultural ecosystems (1). In this partnership, rhizobia convert atmospheric nitrogen into ammonia that the host plants can use for growth, while the plants supply rhizobia with carbohydrates and a protective environment to support their survival and activities (2–4). The initiation of nodulation begins when root-secreted flavonoids attract rhizobia and stimulate them to secrete lipo-chitooligosaccharidic nodulation (Nod) factors (NFs), which are then recognized by root NF receptors (NFRs) to trigger rhizobial infection—a critical step for nodule formation (4).

To prevent excessive nodulation that would increase energy costs and offset symbiotic benefits, legumes have evolved strategies, including autoregulation of nodulation (AON) under low nitrogen (N) conditions and nitrogen regulation of nodulation (NON), to control nodule numbers (5–7). Rhizobia stimulate legume roots to produce CLAVATA3 (CLV3)/EMBRYO SURROUNDING REGION-related (CLE) peptides from posttranslational modification of short prepropeptide precursors. The CLE peptides, such as CLE12 and CLE13 in *M. truncatula*, LjCLE-RS1, LjCLE-RS2, and LjCLE-RS3 in *L. japonicus*, and GmRIC1 and GmRIC2 in soybean, are then transported to shoots to repress the production of family members of microRNA (miRNA) miR2111, which undergo shoot-to-root transportation. Consequently, the reduced accumulation of miR2111 leads to increased expression of their direct targets—symbiosis suppressors *TOO MUCH LOVE*s (*TML*s)—in roots (8–10), which inhibit nodule formation. Considering that early responses of the miR2111-*TML* modules, as indicators of AON-mediated repression of rhizobial infection, are not detectable until approximately 2 to 5 d post inoculation (dpi) with rhizobia, the timeframes before AON activation represent windows during which the plants maintain a susceptible state necessary for rhizobial infection (8–10).

Despite its criticality, this susceptible state alone does not appear sufficient to form an optimal number of nodules. In soybean-rhizobium symbiosis, rhizobial tRNA-derived fragments are secreted into the host cells and hijack the host RNA-interference machinery to silence host genes as early as 6 h post inoculation (hpi), promoting rhizobial infection (11). In *M. truncatula*, C-TERMINALLY ENCODED PEPTIDE 7 (MtCEP7), derived from posttranslational modification of its prepropeptide precursor, is rapidly induced in roots by rhizobia and translocated to shoots to activate a systemic shoot-to-root signaling pathway that promotes nodulation (12).

In this study, we unravel a systemic regulatory loop that involves root-derived mobile peptide GmCEP7 as a nodulation activator, a shoot-derived mobile miRNA miR4416-5p as a nodulation repressor, and a vegetative lectin gene *GmLe3* as a nodulation activator in soybean. We demonstrate how the GmCEP7-miR4416-5p-*GmLe3* loop promotes rhizobial infection to boost soybean plant productivity under limited nitrogen conditions. Comparative genomics analysis reveals the presence of *MIR4416*, the precursor gene of miR4416-5p, and *GmLe3* orthologs in other legume crops, including common bean (*P. vulgaris*) and pigeonpea (*Cajanus cajan*), but the absence of the *MIR4416* in *M. truncatula* and *L. japonicus*, highlighting an evolutionary innovation of systemic nodulation regulation that arose prior to the divergence between these crops and the two model species from a common ancestor.

## Results

### Shoot-Derived miR4416-5p Regulates GmLe3 Expression in Roots.

Using a soybean-common bean grafting system, we identified dozens of shoot-to-root trafficking microRNAs (miRNAs) and variants (13). Among these, the 21-nt soybean miRNA previously annotated as gma-miR4416b showed greatly reduced abundance in developing nodules relative to roots, while its predicted target gene *Glyma.02G156800*, previously known as *Lectin 3* (14) (*GmLe3*), was substantially upregulated in nodules compared to roots (*SI Appendix*, Table S1). *GmLe3* belongs to the legume lectin gene family, which comprises 94 members predicted to encode proteins each harboring a carbohydrate recognition domain (CRD), in the reference genome (15). Legume lectins have long been implicated in nodulation (16); for example, soybean root exudate lectins were found to promote nodulation (17), whereas common bean seed lectins were shown to enhance infection thread formation (18). We therefore further investigated the potential role of the predicted gma-miR4416b-*GmLe3* module in regulating nodulation.

According to miRBase (19), gma-miR4416b is produced from its precursor *gma-MIR4416b* located on chromosome 3 of the soybean reference genome (20). However, *gma-MIR4416b* was not expressed in any examined tissues, including developing roots, cotyledons, leaves, and shoot apexes from Williams 82 seedlings at the second vegetative (V2) stage, when the second trifoliolate is fully opened (*SI Appendix*, Fig. S1*A*). To identify the gma-miR4416b precursor, we screened the reference genome using the gma-miR4416b sequence and found only one additional, perfect match in the genome, which is located on chromosome 19, 70 nt away from the counterpart of gma-miR4416a within its annotated precursor *gma-MIR4416a* (*SI Appendix*, Fig. S1*B*). The shoot tissues were then used to obtain the primary transcript of *gma-MIR4416a* by 5′ and 3′ rapid amplification of cDNA ends (RACE), followed by sequencing of the RACE products (*SI Appendix*, Fig. S1*C*). The full-length transcript from *gma-MIR4416a* and its predicted secondary structure suggests that gma-miR4416b and gma-miR4416a are the miRNA pairs derived from the 5p and 3p arms of the same pri-miRNA. They are thus redesignated (*SI Appendix*, Fig. S1*D*), according to standard nomenclature (21), as miR4416-5p and miR4416-3p, respectively. Correspondingly, their encoding gene is redesignated as *MIR4416*.

**Fig. 1.**
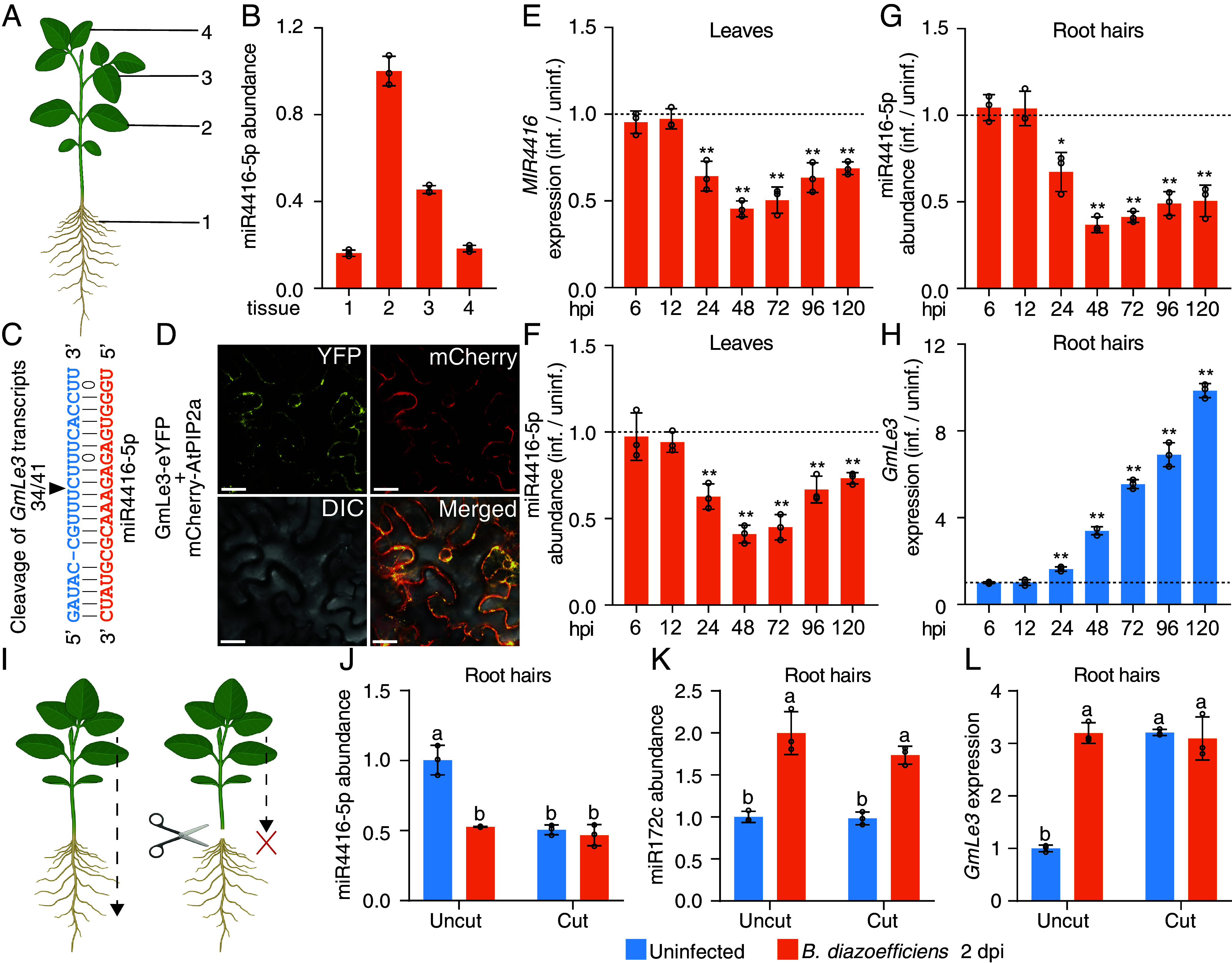
Shoot-derived miR4416-5p regulates the *GmLe3* expression in roots. (*A*) Tissue samples. Roots (1), unifoliate leaves (2), first trifoliate leaves (3), and second trifoliate leaves (4). (*B*) The relative abundances of miR4416-5p in different tissues of the wild type (WT) plants (*n* = 3). The numbers below the *x*-axis denote the corresponding tissues shown in (*A*). The data are reported as the mean ± SD. (*C*) miR4416-5p, its putative target transcript *GmLe3* and the cleavage site and the frequency of 5′ RACE clones (indicated by arrow and ratio) detected in the V2-stage uninoculated roots. (*D*) Subcellular localization of GmLe3. The GmLe3 fusion construct was expressed in *Nicotiana benthamiana* leaves, and its corresponding transient expression was detected by confocal laser scanning microscopy. The “YFP” panel (yellow) represents signal from GmLe3-fused enhanced yellow fluorescent protein; the “mCherry” panel (red) represents signal from plasma membrane-targeted mCherry marker protein, AtPIP2a; the “Merged” panel shows merged YFP and mCherry signals. (Scale bar, 20 µm.) (*E*–*H*) *MIR4416* expression levels (*E*), miR4416-5p abundances (*F* and *G*) and *GmLe3* expression levels (*H*) in infected (inf.) and uninfected (uninf.) plants (*n* = 20) with *Bradyrhizobium diazoefficiens*. Statistical differences were determined with Student’s *t* test. *P* values: *P ≤ 0.05; **P ≤ 0.01. The experiment was repeated three times independently. The data are reported as the mean ± SD. hpi, hours post inoculation. (*I*) Cutting (×) inhibits the downward flow (dashed arrows) of miR4416-5p. (*J*) Root/shoot separation (cutting) reduced miR4416-5p abundances in root hairs, but *B. diazoefficiens* infection triggered miR4416-5p reduction only in intact plants. (*K*) Abundances and infection-responsiveness of miR172c were unaffected by cutting. (*L*) *GmLe3* levels in same samples as in (*J* and *K*). (*J*–*L*) *n* = 20. Statistical differences were determined via ANOVA with a post hoc Tukey test and are indicated by different letters. The experiment was repeated three times independently. dpi, days post inoculation. The data are reported as the mean ± SD. Figure created with BioRender.com.

In the V2-stage, from soybean shoots of the seedlings not inoculated with rhizobia (Fig. 1*A*), stem-loop-PCR detected miR4416-5p at the highest levels in the unifoliate leaves, with gradually decreasing abundance along the first trifoliate leaves and second trifoliate leaves (Fig. 1*B*). As expected, miR4416-5p was detected in roots, but its abundance was lower than that in leaves (Fig. 1*B*). 5′ RACE analysis with the root tissue reveals that *GmLe3* was predominantly cleaved at the predicted miR4416-5p target site (Fig. 1*C*), validating that *GmLe3* was down-regulated by miR4416-5p posttranscriptionally. The GmLe3 protein is localized at the plasma membrane (Fig. 1*D*).

### The miR4416-5p-GmLe3 Module Promotes Rhizobial Infection Systemically Under Low N.

To determine whether the miR4416-5p-*GmLe3* module is involved in initiation of nodulation, we first inoculated the roots of the V2-stage plants with the *B. diazoefficiens* strain USDA110 and examined the responses of the miR4416-5p-*GmLe3* module in the first trifoliate leaves and root hairs. To prevent the negative effects of high N concentrations on rhizobia infection, the experiments were conducted under low N conditions. The expression level of *MIR4416* in leaves started to respond negatively to rhizobia as early as 24 hpi (Fig. 1*E*). As expected, the abundance of miR4416-5p in leaves and root hairs displayed a rhizobium-responsive pattern (Fig. 1 *F* and *G*). This is consistent with the changes in the expression level of *MIR4416* in leaves. In contrast, the *GmLe3* expression in root hairs responded positively to the rhizobia (Fig. 1*H*).

In line with its shoot-to-root mobility, miR4416-5p showed reduced abundance in roots physically separated from shoots (Fig. 1*I*) compared to roots of intact plants with inoculation (Fig. 1*J*). In contrast, miR172c, which is known to be produced locally, did not exhibit a difference in abundance between these two sets of roots (Fig. 1*K*). As expected, miR172c, whose abundance in soybean roots has been demonstrated to respond positively to rhizobia (22), showed a similar level of increases in abundance in shootless roots and roots of intact plants inoculated immediately after shoot-root separation (Fig. 1*K*), indicating that miRNA biogenesis in roots was not impaired over the time of separation. However, the responses of the miR4416-5p-*GmLe3* module to rhizobia in roots of intact plants were not detected in shootless roots (Fig. 1 *J* and *L*), suggesting that it is shoot-derived miR4416-5p that responds to rhizobia to regulate *GmLe3* expression in roots.

To examine potential effects of the miR4416-5p-*GmLe3* module on nodulation, we initially developed soybean transgenic hairy roots expressing artificial miRNA (amiR4416-5p) (*SI Appendix*, Fig. S2*A*) as well as short tandem target mimics (STTM) transgenic hairy roots repressing levels of miR4416-5p (*SI Appendix*, Fig. S2*B*), each under the control of the cauliflower mosaic virus (CaMV) 35S promoter. The STTM method employs a mimic to not just passively block a targeted miRNA, but to induce active degradation of it by the SMALL RNA DEGRADING NUCLEASE, producing phenotypes comparable to a miRNA knockout (23). The plants with hairy roots, and empty vector-transformed hairy roots as the control, were grown in pots with sterilized vermiculite and inoculated with USDA110. Noticeably, compared to the control, the plants with amiR4416-5p roots displayed reduced plant growth with paler leaves and fewer nodules, whereas those with miR4416-5p STTM roots showed enhanced plant growth with darker leaves and more nodules, when examined 28 dpi (*SI Appendix*, Fig. S3).

**Fig. 2.**
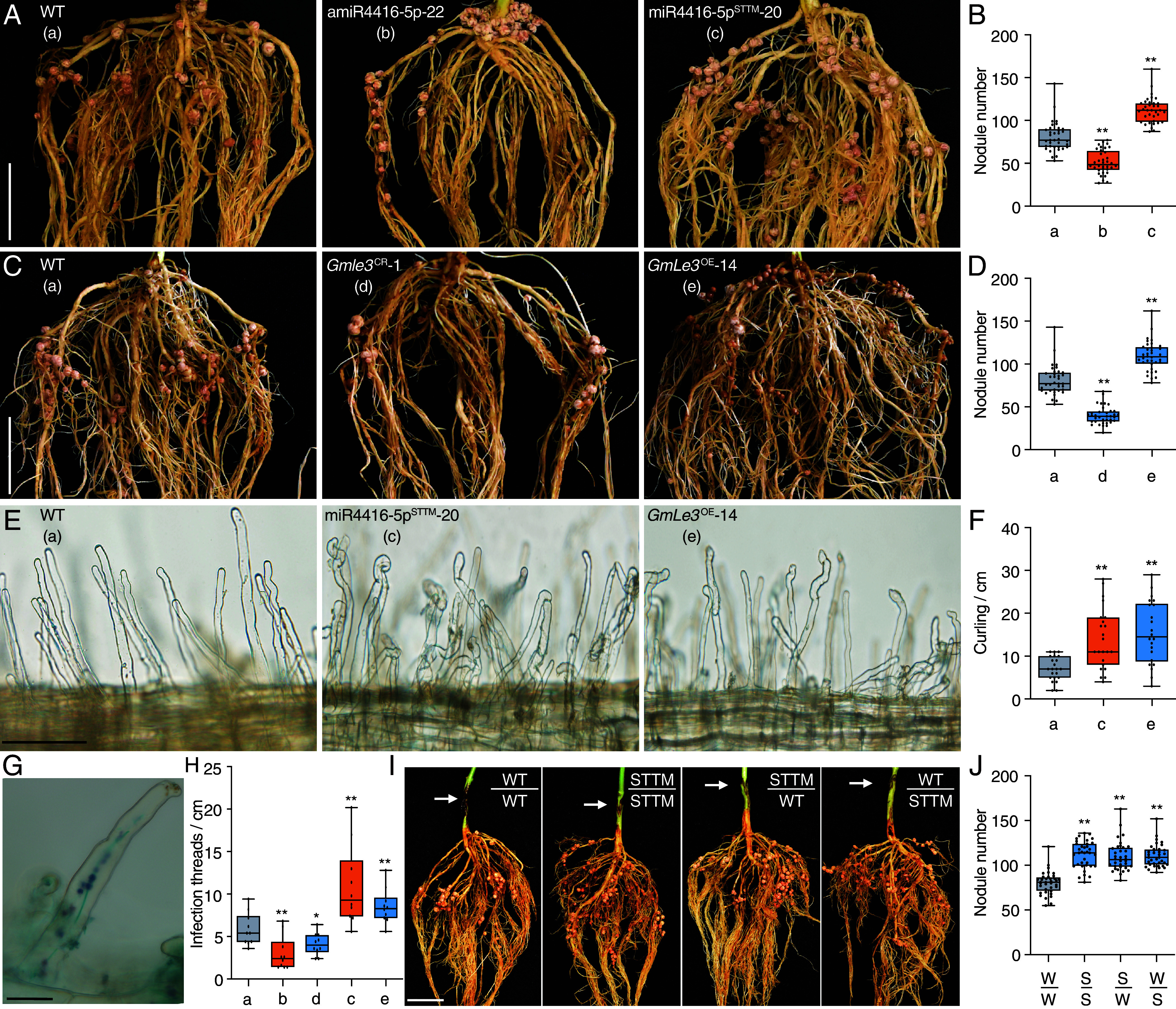
The miR4416-5p-*GmLe3* module promotes rhizobial infection systemically under low nitrogen. (*A*–*D*) Representative images and quantification of nodule numbers (28 dpi) of stable amiR4416-5p and miR4416-5p^STTM^ plants compared with the WT, and stable *Gmle3* mutants and *GmLe3*-overexpression (*GmLe3*^OE^) plants compared with the WT. For all box-and-whisker plots presented in this study, the lines represent the median, the dots represent the data points (*n* = 36), the edges of the boxes define the interquartile ranges, and the whiskers represent the minimum and maximum values. The letters below the *x*-axis denote the corresponding materials shown in (*A*) and (*C*), respectively. Statistical differences were determined with Student’s *t* test. *P* values: **P ≤ 0.01. The experiment was repeated three times independently. (Scale bar, 5 cm.) (*E* and *F*) Representative images and quantification of numbers of curled root hairs (24 hpi) in stable miR4416-5p^STTM^ and *GmLe3*^OE^ plants compared with the WT. WT, *n* = 22; miR4416-5p^STTM^-20, *n* = 23; *GmLe3*^OE^-14, *n* = 22. The letters below the *x*-axis denote the corresponding materials shown in (*E*). Statistical differences were determined with Student’s *t* test. *P* values: **P ≤ 0.01. The experiment was repeated three times independently. (Scale bar, 100 µm.) (*G* and *H*) Representative images and quantification of numbers of infection threads in stable amiR4416-5p, *Gmle3* mutants, miR4416-5p^STTM^ and *GmLe3*^OE^ plants compared with WT. *n* = 10. The letters below the *x*-axis denote the corresponding materials shown in (*A*) and (*C*). Statistical differences were determined with Student’s *t* test. *P* values: *P ≤ 0.05; **P ≤ 0.01. The experiment was repeated two times independently. (Scale bar, 20 µm.) (*I*) Photographic illustration of the phenotypic changes in the nodule (28 dpi) number of grafted WT, miR4416-5p^STTM^ (STTM), and chimeric plants. The white arrows indicate the grafting sites. (Scale bar, 3 cm.) (*J*) Changes in nodule numbers (28 dpi) of grafted plants as exemplified in (*I*). WT (W) and miR4416-5p^STTM^ (S). *n* = 36. Statistical differences were determined with Student’s *t* test. *P* values: **P ≤ 0.01. The experiment was repeated three times independently.

To further validate the effects observed during hairy root transformation, we developed and examined stable amiR4416-5p and miR4416-5p STTM transgenic lines. Consistent with the hairy root experiments, the stable amiR4416-5p transgenic lines, with substantially elevated miR4416-5p levels and reduced *GmLe3* expression (*SI Appendix*, Fig. S4 *A* and *B*), produced fewer nodules than the WT (Fig. 2 *A* and *B* and *SI Appendix*, Fig. S5*A*). In contrast, the stable miR4416-5p STTM lines, in which miR4416-5p was severely suppressed and *GmLe3* expression was concomitantly increased (*SI Appendix*, Fig. S4 *A* and *B*), developed more nodules (Fig. 2 *A* and *B* and *SI Appendix*, Fig. S5*A*). These lines were planted in Purdue Agronomy farm for seed propagation and evaluation for agronomic traits without the application of any fertilizers. Consistent with the observations from the hairy root experiments, the amiR4416-5p lines exhibited reduced productivity, as reflected by diminished yield component traits including plant height, primary branch number, main stem node number, pod number per plant, and seed number per plant traits (*SI Appendix*, Fig. S5 *B–G*). In contrast, the miR4416-5p STTM lines displayed increased plant productivity (*SI Appendix*, Fig. S5 *B–G*), with two examined lines showing average increases of 15% and 39% in seed number per plant, respectively. In addition, we developed stable *GmLe3*-editing lines and *GmLe3*-overexpression lines under the control of the 35S promoter. *Gmle3*^CR^ mutants with premature stop codons (*SI Appendix*, Fig. S6*A*) produced fewer nodules than the WT (Fig. 2 *C* and *D* and *SI Appendix*, Fig. S6*B*), while *GmLe3*-overexpression lines (*SI Appendix*, Fig. S6*C*) formed more nodules than the WT (Fig. 2 *C* and *D* and *SI Appendix*, Fig. S6*B*).

Given that the miR4416-5p-*GmLe3* module responds to rhizobia as early as 24 hpi, we hypothesized that its regulatory role in nodulation likely involves modulating rhizobial infection. To test this hypothesis, we inoculated the roots of V2-seedlings of the miR4416 STTM-lines and *GmLe3*-overexpression lines, as well as the wild-type controls, and examined root hairs within a 1-cm zone at the top of the primary root of each seedling. While root hair numbers and lengths were similar across all lines, the miR4416-5p STTM-lines and *GmLe3*-overexpression lines had increased numbers of curled and deformed root hairs compared to the WT (Fig. 2 *E* and *F* and *SI Appendix*, Fig. S6 *D* and *E*). We also examined the densities of infection threads (ITs) as detected by GUS-tagged rhizobia. The miR4416-5p STTM lines and *GmLe3*-overexpression lines formed more ITs than the WT. By contrast, the amiR4416-5p and *Gmle3*^CR^ lines formed fewer ITs than the WT (Fig. 2 *G* and *H* and *SI Appendix*, Fig. S6*F*). Together, these results indicate that miR4416-5p functions as a nodulation suppressor, *GmLe3* acts as a nodulation activator, and the miR4416-5p-*GmLe3* module promotes early stage rhizobial infection, resulting in increased nodule number.

Although miR4416-5p is primarily produced in the shoots and translocated to the roots, a small amount, if produced locally in roots, could show some level of effects on nodulation. To evaluate the extent of miR4416-5p-mediated systemic regulation of nodulation, we performed grafting experiments involving four distinct grafted scion/rootstock combinations of the miR4416-5p STTM line (STTM) (line #20) and the WT: WT/WT, STTM/STTM, STTM/WT, and WT/STTM. The grafted plants were inoculated with USDA110 after their recovery (~7 d postgrafting). The STTM/STTM, STTM/WT, and WT/STTM plants all produced more nodules than the WT/WT plants, but no differences were observed between the STTM/WT and the WT/STTM plants or between the STTM/WT and the STTM/STTM plants (Fig. 2 *I* and *J*). These results suggest that miR4416-5p translocated to roots from shoots primarily regulates nodulation.

### Root-Derived GmCEP7 Promotes Nodulation by Repressing miR4416-5p Production in Shoots.

**Fig. 3.**
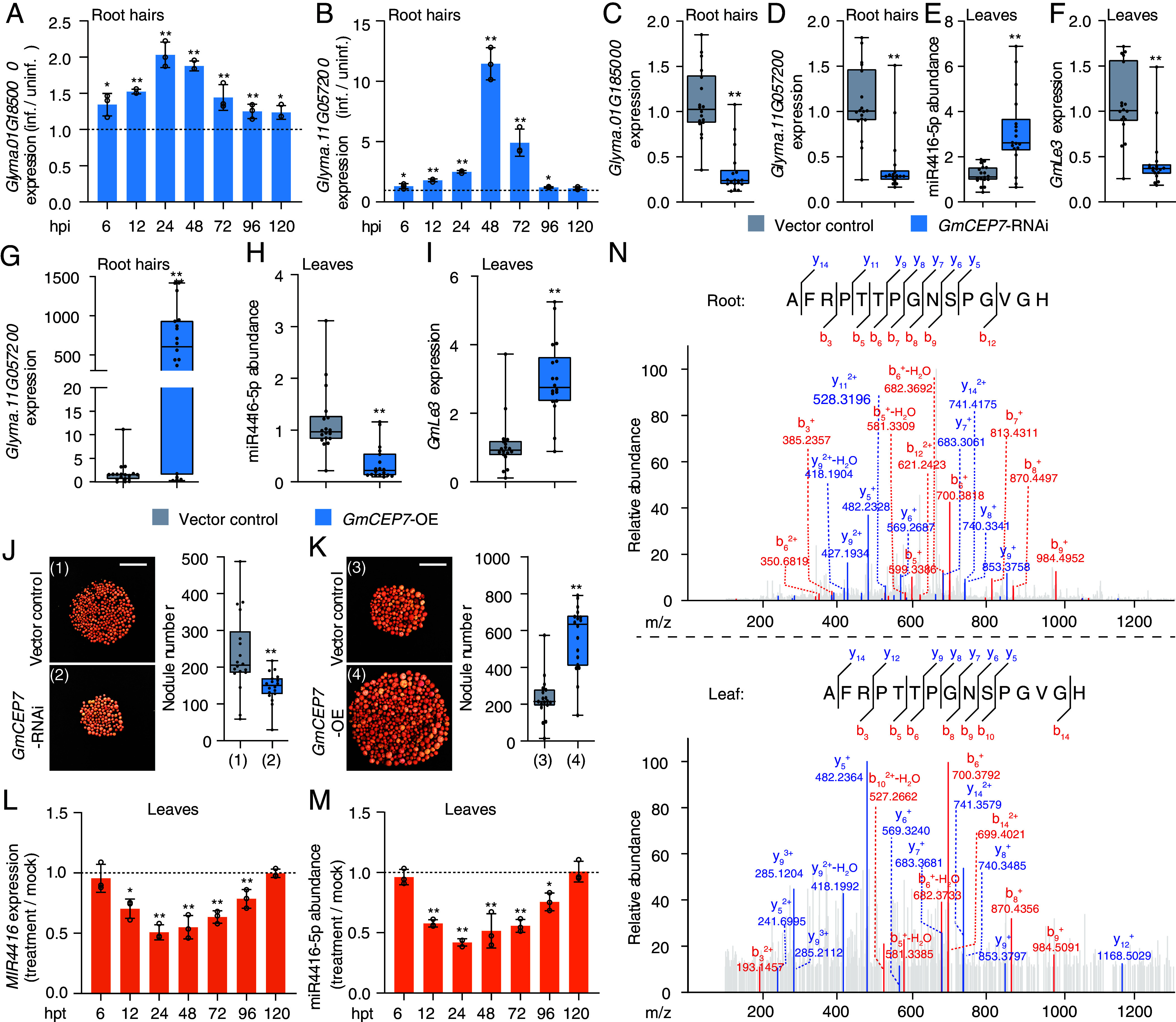
Repression of miR4416-5p biogenesis in shoots by root-derived peptide GmCEP7 promotes nodulation. (*A* and *B*) *Glyma.01G185000* (*A*) and *Glyma.11G057200* (*B*) expression levels in infected (inf.) and uninfected (uninf.) plants (*n* = 20) with *B. diazoefficiens*. Statistical differences were determined with Student’s *t* test. *P* values: *P ≤ 0.05; **P ≤ 0.01. The experiment was repeated three times independently. The data are reported as the mean ± SD. hpi, hours post inoculation. (*C* and *D*) *Glyma.01G185000* (*C*) and *Glyma.11G057200* (*D*) expression levels in *GmCEP7*-RNAi transgenic hairy roots compared with the vector control. For all box-and-whisker plots presented in this study, the lines represent the median, the dots represent the data points (*n* = 18), the edges of the boxes define the interquartile ranges, and the whiskers represent the minimum and maximum values. Statistical differences were determined with Student’s *t* test. *P* values: **P ≤ 0.01. The experiment was repeated three times independently. (*E* and *F*) Abundance/expression of miR4416-5p/*GmLe3* in leaves of the *GmCEP7*-RNAi composite plants compared with the vector control. *n* = 18. Statistical differences were determined with Student’s *t* test. *P* values: **P ≤ 0.01. The experiment was repeated three times independently. (*G*) *Glyma.11G057200* expression levels in *GmCEP7*-OE transgenic hairy roots compared with the vector control. *n* = 18. Statistical differences were determined with Student’s *t* test. *P* values: **P ≤ 0.01. The experiment was repeated three times independently. (*H* and *I*) Abundance/expression of miR4416-5p/*GmLe3* in leaves of the *GmCEP7*-OE composite plants compared with the vector control. *n* = 18. Statistical differences were determined with Student’s *t* test. *P* values: **P ≤ 0.01. The experiment was repeated three times independently. (*J* and *K*) Representative images and quantification of nodule number at 28 dpi in *GmCEP7*-RNAi (*J*) and *GmCEP7*-OE (*K*) composite plants compared with vector controls. *n* = 18. Statistical differences were determined with Student’s *t* test. *P* values: **P ≤ 0.01. The experiment was repeated three times independently. (Scale bar, 2 cm.) (*L* and *M*) *MIR4416* Expression levels (*L*) and miR4416-5p abundance (*M*) in leaves upon GmCEP7 treatment. Deionized water was used as mock control. Statistical differences were determined with Student’s *t* test. *P* values: *P ≤ 0.05; **P ≤ 0.01. The experiment was repeated three times independently. The data are reported as the mean ± SD. hpt, hours post treatment. (*N*) Synthetic GmCEP7 carrying stable-isotope-labeled ^13^C_9_, ^15^N-Phenylalanine and hydroxyprolines modifications (HyP4, HyP7, and HyP11) (hereafter referred to as GmCEP7^iso^) were detected in both roots and leaves of soybean plants following root treatment with GmCEP7^iso^.

C-TERMINALLY ENCODED PEPTIDEs (CEPs) are 15-amino-acid hormones produced in roots, which are derived from posttranslational modification of short prepropeptide precursors encoded by multigene families expressed in roots (24, 25). In *M. truncatula*, MtCEP7 is, to date, the only peptide known to be induced in roots by rhizobia that promotes nodulation systemically (26). Therefore, we wondered whether the counterpart of MtCEP7 in soybean promotes nodulation, and if so, whether it functions through regulating miR4416-5p production in shoots. In soybean, four genes are closely related to the MtCEP7 and MtCEP1 precursors. Their annotated proteins all include the “AFRPTTPGNSPGVGH” sequence that is slightly diverged from MtCEP7/MtCEP1 (*SI Appendix*, Fig. S7*A*). Among these, *Glyma.17G176800* and *Glyma.17G177000* are primarily expressed in stems (*SI Appendix*, Fig. S7 *B* and *C*), while *Glyma.01G185000* and *Glyma.11G057200* are root-specific (*SI Appendix*, Fig. S7 *D* and *E*) and respond positively to rhizobia (Fig. 3 *A* and *B*); these are designated GmCEP7 precursors. The expression of both *Glyma.01G185000* and *Glyma.11G057200* responded to rhizobia as early as 6 hpi, with their responses remaining detectable until 96 hpi (Fig. 3 *A* and *B*).

To understand the role of *Glyma.01G185000* and *Glyma. 11G057200*, we generated soybean transgenic hairy roots in which both genes were knocked down by RNAi (dubbed *GmCEP7*-RNAi), as well as transgenic hairy roots overexpressing *Glyma.11G057200* under the control of the 35S promoter (dubbed *GmCEP7*-OE), with empty vector-transformed hairy roots as corresponding controls. Knockdown of these genes in hairy roots (Fig. 3 *C* and *D*) resulted in increased miR4416-5p abundance (Fig. 3*E*), decreased *GmLe3* expression in the first trifoliate leaves (Fig. 3*F*), and reduced nodule number (Fig. 3*J*). In contrast, overexpression of *Glyma.11G057200* in hairy roots (Fig. 3*G*) resulted in decreased miR4416-5p abundance, elevated *GmLe3* expression in the first trifoliate leaves (Fig. 3 *H* and *I*), and increased nodule number (Fig. 3*K*). These observations suggest that rhizobia-induced, root-specific expression of *Glyma.01G185000* and *Glyma.11G057200* promotes nodulation.

Given the evolutionary and functional conservation between GmCEP7 and MtCEP7 in promoting nodulation, GmCEP7 was hypothesized to be the signaling molecule that regulates *MIR4416* expression in shoots. To test this hypothesis, the synthetic GmCEP7 peptides were applied to the roots of V2-stage soybean seedlings that were not inoculated with rhizobia, and *MIR4416* transcript levels and miR4416-5p abundances were examined in the first trifoliate leaves over a timeframe of 120 h post treatment (hpt) with the peptide. The *MIR4416* transcript levels and miR4416-5p abundances began to respond to GmCEP7, negatively, as early as 12 hpt (Fig. 3 *L* and *M*). These observations suggest that GmCEP7 regulates miR4416-5p-mediated signaling pathways.

To track the root-to-shoot movement of GmCEP7, we employed liquid chromatography–tandem mass spectrometry (LC–MS/MS) to detect and quantify GmCEP7 in roots of wild-type soybean plants as well as in *GmCEP7*-OE transgenic hairy roots (Fig. 3*G*). However, even in the *GmCEP7*-OE hairy roots, in which *GmCEP7* transcript levels increased more than 1,000-fold (Fig. 3*G*), the GmCEP7 peptide remained undetectable. Alternatively, we applied a synthetic, stable-isotope-labeled (^13^C_9_, ^15^N-phenylalanine) GmCEP7 to track its movement. This labeled peptide was applied to the roots of V2-stage wild-type soybean seedlings for 3 d, after which total proteins were extracted separately from roots and leaves and enriched for targeted detection by LC–MS/MS. The labeled GmCEP7 peptide was detected in both roots and leaves (Fig. 3*N* and *SI Appendix*, Fig. S8), validating the mobility of GmCEP7 from roots to shoots.

### miR4416-5p-Mediated Systemic Regulation of Nodulation Marks an Evolutionary Innovation.

**Fig. 4.**
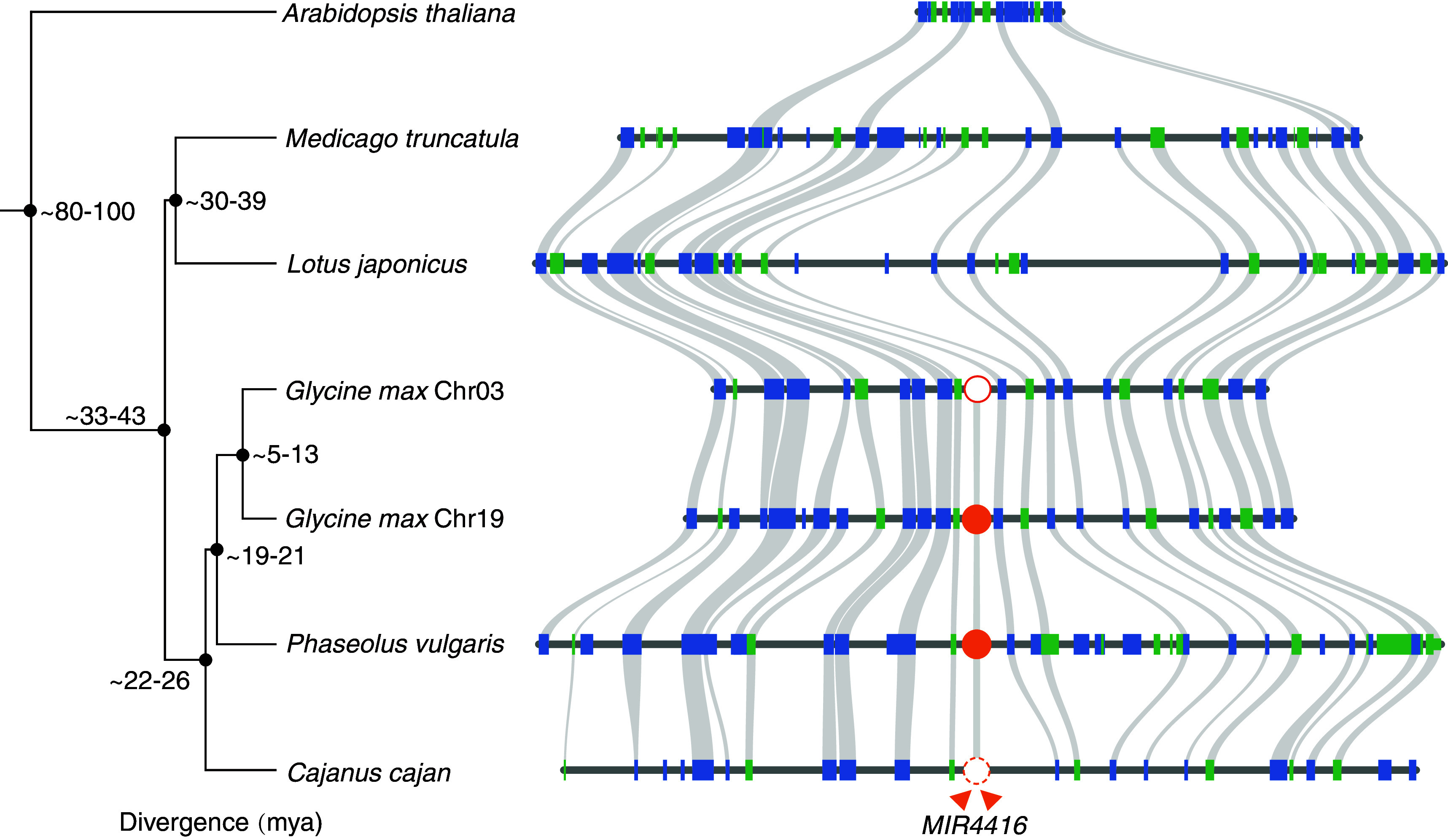
miR4416-5p-mediated systemic regulation marks an evolutionary innovation. The phylogenetic tree illustrates the evolutionary relationships among five representative legume species, with *A. thaliana* as the outgroup. The microsynteny plot displays the *MIR4416* locus from these species. Blue boxes represent genes on the forward strand, while green boxes denote genes on the reverse strand. *MIR4416* location is highlighted by red color. Solid circle: *MIR4416* exists and can be expressed; empty circle: *MIR4416* exists but expression was not detected; dashed empty circle: *MIR4416* exists but no expression data are currently available.

To shed light on evolutionary conservation and divergence of miR4416-5p-mediated systemic regulation of nodulation across species, we conducted a comparative genomics analysis of representative legume species, with Arabidopsis as an outgroup. These include tropical season legumes such as soybean, common bean, and pigeonpea (*C. cajan*) producing determinate nodules, and cool season legumes *M. truncatula* and *L. japonicus* forming indeterminate nodules. Based on the paired stem-loop sequences of *MIR4416* that correspond to miR4416-5p and miR4416-3p, we identified putative *MIR4416* orthologs in common bean and pigeonpea (Fig. 4), although neither has previously been annotated as a miRNA precursor gene. Additionally, abundant miRNAs corresponding to miR4416-5p and miR4416-3p were found in small RNA datasets from common bean (27–29). The putative miR4416-5p in common bean was also predicted to target a *GmLe3* homolog, suggesting that the miR4416-5p-mediated regulation may be conserved among these crops. However, no *MIR4416* orthologs or homologs were found in *M. truncatula* or *L. japonicus*, suggesting these model legumes may employ distinct mechanisms promoting nodulation.

## Discussion

Our study elucidates the GmCEP7-miR4416-5p-*GmLe3* systemic regulatory loop that promotes nodulation in soybean. Although lectins are widely regarded as contributors to rhizobial recognition, attachment, and early signaling events that lead to nodulation (16, 30, 31), how symbiotic signals and systemic pathways regulate lectins have not been previously known or validated in vivo. This study thus provides a mechanistic understanding regarding spatial, temporal, and pathway-level regulation of a lectin protein in symbiotic signaling.

Lines of experimental evidence strongly support GmCEP7 as a root-derived mobile signal that regulates miR4416-5p biosynthesis in shoots: 1) synthetic GmCEP7 applied to roots reduces miR4416-5p production in leaves (Fig. 3*M*); 2) rhizobia induce the expression of putative GmCEP7 precursor genes (Fig. 3 *A* and *B*); 3) Silencing these precursor genes in roots increases miR4416-5p production in leaves (Fig. 3 *C*–*E*); 4) Overexpressing a GmCEP7 precursor gene in roots reduced miR4416-5p production in leaves (Fig. 3 *G* and *H*); and 5) Stable-isotope-labeled GmCEP7 applied to roots was directly detected in leaves (Fig. 3*N* and *SI Appendix*, Fig. S8). These observations align with the validated movement and function of MtCEP7 in *M. truncatula*, highlighting the evolutionary conservation of CEP peptides-mediated root-shoot communications in legumes. The root-specific expression of *Glyma.01G185000* and *Glyma.11G057200* suggests that they are the precursors of the GmCEP7 peptide in roots, but how *Glyma.17G176800* and *Glyma.17G177000* have functionally diverged from the two *GmCEP7* genes, as reflected by their predominant expression in stems and lack of expression in roots, remains unclear.

The absence of miR4416-5p in *M. truncatula* does reflect a remarkable distinction in CEP7-mediated regulation of nodulation between these species. Therefore, it is important to integrate knowledge from model legumes and crop species to better understand the regulatory mechanisms underlying this biological process vital for sustainable agriculture. Although comparative genomics suggests the presence of the *MIR4416* and *GmLe3* orthologs in two additional economically important legume crops, common bean and pigeonpea, the conservation and functionality of these genes remain to be systematically investigated.

We would note that most molecular assays were performed at early plant developmental stages with a limited number of mature leaves—where the most abundant miR4416-5p was produced—to comply with growth space limits and to increase plant sample sizes. Thus, the magnitude of miR4416-5p-mediated systemic regulatory effects may have been underestimated. In addition, given the continuous nature of plant growth and nodulation, the GmCEP7-miR4416-5p-*GmLe3* regulatory loop likely coordinates dynamically with other mechanisms to optimize nodule numbers, balancing the benefits of symbiosis against its metabolic costs. Apparently, such benefits are reflected by architectural changes such as increased branch number, node number, and plant height, in addition to increased pod and seed numbers per plant, as observed in the miR4416-5p STTM lines. These changes are consistent with previously reported yield increases resulting from partial AON impairment under low N conditions (32). However, whether genetic modification of the GmCEP7-miR4416-5p-*GmLe3* module can enhance photosynthesis efficiency and ultimately improve crop yields remains to be determined through comprehensive field evaluations under various conditions.

## Materials and Methods

Detailed information on plant growth and bacteria inoculation, sRNA-seq and mRNA-seq data analysis, miRNA abundance and gene expression analysis, miRNA target site prediction and target site validation, vector construction and plant transformation, subcellular localization assays, grafting, peptides treatment, stable isotope labeled synthetic peptides tracking, and syntenic analysis of the *MIR4416* loci are provided in *SI Appendix*, *Materials and Methods*. The primers used in this study are listed in *SI Appendix,* Table S2.

## Supplementary Material

Appendix 01 (PDF)

## Data Availability

All study data are included in the article and/or *SI Appendix*.
