# Supplementary material for: A long-distance signaling loop promotes soybean nodulation and productivity
Source: Proc Natl Acad Sci U S A. 2026 Jun 8;123(24):e2609325123. doi: 10.1073/pnas.2609325123 (PMC13273300; doi:10.1073/pnas.2609325123)
Supplement: Supplementary file 1 — Appendix 01 (PDF) [file pnas.2609325123.sapp.pdf]

PNAS

Supporting Information for

A long-distance signaling loop promotes soybean nodulation and productivity

Jingbo Duan[a,b,1], Jinbin Wang[a,b,1], Runze Guo[a,b,1], Chancelor B. Clark[a,b], Zhuojun Luo[c], Xiaochong Li[a,b], Leonie Trabert[a,b], Xing-Qi Huang[b,c], W. Andy Tao[c], Natalia Dudareva[b,c], Gary Stacey[d], Blake C. Meyers[e], Jianxin Ma[a,b]. *[1]J.D., J.W., and R.G. contributed equally to this work.*

Corresponding authors:

Blake C. Meyers (email: bcmeyers@ucdavis.edu) and Jianxin Ma (email: maj@purdue.edu)

[a] Department of Agronomy, Purdue University, West Lafayette, IN 47907, USA
[b] Center for Plant Biology, Purdue University, West Lafayette, IN 47907, USA
[c] Department of Biochemistry, Purdue University, West Lafayette, IN 47907, USA
[d] Division of Plant Science & Technology, University of Missouri, Columbia, MO 65211, USA
[e] Genome Center and Department of Plant Sciences, University of California, Davis, CA 95616, USA

This PDF file includes:

Supporting text
Figures S1 to S8
Tables S1 to S2
SI References

Supporting Information Text

Materials and Methods

Plant and bacterial resources. The soybean (*Glycine max*) cultivar Williams 82 (1) was used as the wild type. Plants were infected with *Bradyrhizobium diazoefficiens* USDA110 or GUS-tagged *B. diazoefficiens* USDA110 bacteria. Cloning was performed using DH5α. *Agrobacterium tumefaciens* EHA105 was used for whole plant transformation and *Agrobacterium rhizogenes* K599 for hairy root induction.

Plant growth and inoculation. Soybean seeds were surface sterilized overnight with chlorine gas (2) at room temperature. For growth in low-nitrogen medium (Fig. 1 *B* and *C*, 1 *E-H*, 1 *J-L*, 2 *A-J*, 3*A*, 3*B*, 3 *L-N*, *SI Appendix*, Fig. S1 *A* and *C*, S4 *A* and *B*, S5*A*, S6 *B-F*, S7 *B-E*, and S8), sterilized seeds were sown, germinated, and grown in sterilized vermiculite (Greenhouse Megastore, US) under 16 h light/8 h dark cycles at 25°C (day)/20°C (night) before rhizobium inoculation. From the time of sowing, plants were irrigated with a 500 μM nitrate solution (NS) containing 500 μM NH_4NO_3, 2500 μM $CaCl_2$, 1500 μM K_2SO_4, 1000 μM $MgCl_2$, 0.38 μM $ZnCl_2$, 1.57 μM $CuCl_2$, 0.09 μM $(NH_4)_6Mo_7O_4$, 23.13 μM H_3BO_3, 4.57 μM $MnCl_2$, 250 μM KH_2PO_4, 100 μM Na_2EDTA and 100 μM $FeCl_2$ (pH5.8). For inoculation, *B. diazoefficiens* was cultured in a modified arabinose gluconate (MAG) medium (3) for 3 days and harvested by centrifuging for 10 min at 4,629 × *g*. The bacterial pellet was washed twice with 500 μM NS and diluted to an optical density at λ=600 (OD_{600}) of 0.05. A 20-mL bacterial suspension was applied to each root. Uninfected roots were mock-treated with an equal amount of 500 μM NS without rhizobia. For time-course experiments (Fig. 1 *E-H*, 3 *A*, *B*, *L* and *M*), plants within an experiment were harvested simultaneously following consecutive infection events at appropriate time points and thus had the same age at harvest. Root hair cells were harvested from the roots following a protocol described previously (4). For the shoot-root separation analysis (Fig. 1 *J-L*), 14-day-old seedlings were cut at the primary root initiation site below the hypocotyl, and the remaining shootless root systems were retained in vermiculite. Roots were infected or mock-treated (uninfected control) immediately after cutting.

For determination of gene expression levels and phenotyping assays of composite plants composed of transgenic roots and wild-type shoots (Fig. 3 *C-K*), soybean hairy root transformation was performed as previously described (5) with minor modifications. The seed coat and roots were removed from 4-day-old soybean seedlings, and explants with about 5 mm of hypocotyl were placed in a suspension of *A. rhizogenes* harboring the construct of interest for 30 min. Next, 15 to 20 explants were placed on filter paper pre-wetted with liquid co-cultivation medium and cultured for 4 days. After co-cultivation, the explants were transferred onto filter paper pre-wetted with deionized water. After 4 days, explants with elongated stems and expanded hypocotyls were selected and cultivated in 500 μM NS. After one week, successful transformation was confirmed by the appearance of red pigmentation driven by the *RUBY* (6) visible reporter. Each plant with 3 positive transgenic roots was transplanted into sterilized vermiculite and irrigated with 500 μM NS for 4 days under 16 h light/8 h dark cycles at 25°C (day)/20°C (night) before rhizobium inoculation. For inoculation, liquid cultures of *B. diazoefficiens* were grown and prepared as described above and applied directly to the root systems. The phenotypes were investigated after 4 weeks. Unless otherwise stated, nodules were harvested and counted at 28 dpi from the plants.

sRNA-seq and mRNA-seq data analysis. Soybean shoot-to-root mobile small RNAs (sRNAs) were characterized previously (7). The sRNA-seq and mRNA-seq data were obtained from uninoculated soybean roots, 10 dpi nodules, and 20 dpi nodules, as generated in our previous study (8) and available in NCBI (PRJNA495327).

To validate the production of miRNAs corresponding to miR4416-5p in common bean, the published raw sRNA-seq data were processed using the fastx-toolkit (v.0.0.14, http://hannonlab.cshl.edu/fastx_toolkit) to remove low-quality reads and reads shorter than 17 nt

or longer than 25 nt. The processed reads were aligned to the *Phaseolus vulgaris* genome (v2.1, available at http://phytozome.jgi.doe.gov/) using the Bowtie program (9) (v.1.3.1) with 0 mismatches (−v 0). The abundance of miRNAs corresponding to miR4416-5p in common bean was quantified based on these perfectly mapped reads.

DNA and RNA isolation, PCR, and sequencing. Genomic DNA isolation, PCR, qRT-PCR, and sequencing of DNA fragments and PCR products were performed as previously described (10). Gene expression was measured by qRT-PCR, in which the soybean gene *GmELF1b* (*Glyma.02G276600*) was used as a reference gene to quantify the relative expression levels of the soybean transcripts from three biological replicates. Stem-loop RT-qRT-PCR (11) was performed to evaluate the relative abundance of miR4416-5p in three biological replicates. The specificity of stem-loop RT-qRT-PCR was confirmed by sequencing the amplified fragments. Primers used for the various PCR assays are listed in *SI Appendix*, Table S2.

Full-length transcript cloning. Normal or nested 5′ and 3′ RACE products were obtained using the GeneRacer™ Kit (Invitrogen, MA) following the manufacturer's protocol. Briefly, DNase-treated total RNA from shoot tissues of uninoculated Williams 82 plants was treated with calf intestinal phosphatase (CIP) to remove all active 5′ monophosphates from truncated or otherwise degraded mRNA as well as non-mRNA, rendering those RNAs unavailable for ligation and leaving only intact capped mRNA unaffected. Subsequently, tobacco acid pyrophosphatase (TAP) was used to remove the 5′ cap structure, thereby generating a unique active 5′ phosphate on mature mRNA through hydrolysis of the pyrophosphate bonds in the m_7G cap triphosphate bridge. GeneRacer™ RNA Oligo was ligated to the unique active 5′ phosphate using a T4 RNA ligase in a 10 µL reaction containing dephosphorylated, decapped RNA and 0.25 µg GeneRacer™ RNA Oligo, 1× Ligase Buffer, 1 mM ATP, 40 U RNaseOUT™ and 5 U T4 RNA ligase. First-strand cDNA was obtained by reverse transcription of the ligated mRNA using the GeneRacer™ Oligo dT Primer. For 5' RACE, two rounds of PCR amplification were performed with forward primers located within the GeneRacer™ RNA Oligo (GeneRacer™ 5' Primer and GeneRacer™ 5' Nested Primer) and reverse primers in target transcript (*SI Appendix*, Table S2). For 3' RACE, two rounds of PCR amplification were performed with forward primers located within target transcript (*SI Appendix*, Table S2) and reverse primers in GeneRacer™ Oligo dT (GeneRacer™ 3' Primer and GeneRacer™ 3' Nested Primer). Nested PCRs were performed using a 1:50 dilution of the first round PCR product as a template. The PCR products were cloned into the pCR™4Blunt-TOPO™ vector (Invitrogen, MA) and sequenced at Eurofins Genomics (Louisville, KY) to experimentally confirm the transcription start sites (TSSs) and transcription termination sites (TTSs) that define the full-length transcripts.

RNA secondary structure prediction. The secondary structure of the primary transcript of gma-miR4416a/b was predicted using the RNAfold server incorporated in the ViennaRNA Web Services (http://rna.tbi.univie.ac.at/).

miRNA target site prediction and target site validation. psRNATarget, a plant small RNA target analysis server (12), was used to predict target genes of miR4416-5p based on plant miRNA targeting rules. *Glyma.02G156800* (*GmLe3*) was predicted as a strong candidate based on the following criteria: 1) the expectation value determined by the server was no more than 4; 2) the expression levels displayed at least an 18-fold increase in 10 dpi and 20 dpi nodules compared to those in uninoculated roots.

The miR4416-5p-guided cleavage site in *GmLe3* was identified by 5' RNA ligase-mediated rapid amplification of cDNA ends (RLM-RACE) using the GeneRacer™ Kit (Invitrogen, MA) following the manufacturer's protocol. Briefly, DNase-treated total RNA isolated from root hairs of uninoculated Williams 82 plants was used as input. GeneRacer™ RNA Oligo was ligated to RNAs carrying a 5′ monophosphate, including truncated mRNAs and non-mRNA species, thereby excluding intact capped full-length mRNAs from subsequent reverse transcription. First-strand cDNA was obtained by reverse transcription of the ligated RNA using the GeneRacer™ Oligo dT Primer. The cDNA samples were amplified by nested PCR. Two rounds of PCR amplification were

performed with forward primers located within the GeneRacer™ RNA Oligo (GeneRacer™ 5' Primer and GeneRacer™ 5' Nested Primer) and reverse primers near the end of the 3' untranslated region of *GmLe3* (*SI Appendix*, Table S2). Then the PCR products were cloned into the pCR™4Blunt-TOPO™ vector (Invitrogen, MA) and sequenced at Eurofins Genomics (Louisville, KY) to determine the distribution and frequencies of cleaved fragments that define miR4416-5p-guided cleavage sites within the predicted target *GmLe3*.

Vector construction and stable transformation of soybean. For the subcellular localization of GmLe3, the full-length *GmLe3* coding sequence (CDS) was amplified by the primers listed in *SI Appendix*, Table S2 and then cloned into the binary vector pCNHP-eYFP digested with *Nco*I, which expresses a fusion protein with a C-terminal enhanced yellow fluorescent protein (eYFP).

For all the vectors used for hairy root induction, the *Basta resistance* (*bar*) gene cassette in binary vector pPTN1171 (13) was replaced by the *RUBY* cassette to generate the parent vector pRUBY. To develop miR4416-5p short tandem target mimic (STTM) (14) and artificial microRNA (amiR) (15) vectors, the primer pairs listed in *SI Appendix*, Table S2 were held at 95℃ for 4 min and then cooled to 16℃ to form dimers, which were then inserted into the *Sac*I- and *Xba*I-digested pRUBY vector, separately. To construct the *GmCEP7*-RNAi vector, an intron from the *chalcone synthase A* gene, flanked by two added multiple cloning sites (MCSs) at each end, was cloned into pRUBY to generate the pRUBY-RNAi vector. A general strategy for constructing an RNAi vector involved subcloning an inverted repeat into pRUBY-RNAi at two MCSs. The siRNA sequence was selected using the SGN VIGS TOOL (https://vigs.solgenomics.net/) to ensure that it silenced *Glyma.01G185000* and *Glyma.11G057200* simultaneously while lacking significant sequence homology with other genes. Insertion of the individual repeat fragments into pRUBY-RNAi was achieved in two separate steps. A 300 bp fragment corresponding to nucleotides 146-445 of the *Glyma.01G185000* coding region was amplified by primer pair 1 listed in *SI Appendix*, Table S2 and integrated into the pRUBY-RNAi vector digested with *Asc*I. The inverted fragment was then amplified by primer pair 2 listed in *SI Appendix*, Table S2 and integrated into the *Xba*I site of the intermediate vector to generate *GmCEP7*-RNAi. To construct the *GmCEP7*-OE vector, the *Glyma.11G057200* CDS was amplified by the primers listed in *SI Appendix*, Table S2 and then inserted into *Asc*I- and *Xba*I-digested pRUBY-RNAi. Only root systems with >90% of roots showing ruby color were considered in the analysis. To detect gene expression levels and investigate phenotypes simultaneously, composite plants carrying hairy roots induced by the empty, *GmCEP7*-RNAi, or *GmCEP7*-OE vectors were divided into two groups. Each group had the same number of plants in each biological replicate. One group without rhizobia inoculation was used for tissue collection and determination of gene expression levels (Fig. 3 *C*-*I*). The other group with rhizobia inoculation was used for phenotyping (Fig. 3 *J* and *K*).

The binary vector pEGAD was used to develop miR4416-5p STTM and amiR constructs. The primer pairs containing the STTM and amiR modules were annealed to form dimers, and then inserted into the *Age*I- and *Bam*HI-digested pEGAD vector, separately, to form the constructs.

The *GmLe3* CDS was amplified by the primers listed in *SI Appendix*, Table S2 and then integrated into the pPTN1171 digested by *Nco*I and *Xba*I to develop the overexpression vector. The pPTN1171 contains the cauliflower mosaic virus (CaMV) 35S promoter, the tobacco etch virus translational enhancer upstream of the *Nco*I site and the 35S termination sequence downstream of the *Xba*I site to facilitate expression in plant cells.

The pGES201 vector was used to develop a gRNA-Cas9 expression vector following a protocol described previously (16). An sgRNA for editing the *GmLe3* gene was designed using CRISPRdirect, a web-based guided RNA design software (17). The primer pair listed in *SI Appendix*, Table S2 was annealed using the same setup as for the STTM vectors and then inserted into the *Bsa*I-digested pGES201 vector. To understand the mutations caused by CRISPR/Cas9 at the transcriptional level, we performed RT-PCR followed by Sanger sequencing on stable transgenic lines. We found two types of small deletions in *GmLe3* mRNA (*SI Appendix*, Fig. S6*A*). One type is the deletion of one nucleotide (5'-G-3') corresponding to nucleotide 289 of

the *GmLe3* coding region, which resulted in a frameshift and premature protein termination, producing a truncated 140-amino-acid protein instead of the 282-amino-acid wild-type protein. The other type is the substitution and deletion of three nucleotides (from 5'-GGG-3' to 5'-TT-3') corresponding to nucleotides 287-289 of the *GmLe3* coding region, which resulted in a frameshift and premature protein termination, producing a truncated 140-amino-acid protein instead of the 282-amino-acid wild-type protein.

After their correctness was confirmed by Sanger sequencing, the above-mentioned vectors except those used for hairy root induction were transformed into *A. tumefaciens*. The stable transgenic soybean plants were generated via the *A. tumefaciens*-mediated cotyledonary node protocol (18). Williams 82 was used as the transformation recipient and as the wild type for phenotyping. Transgenic plants were identified by PCR of unique sequences from respective vectors and further validated by sequencing of PCR fragments.

Transgenic line phenotyping in the field. The yield components (plant height, primary branch number, main stem node number, pod number, and seed number) were examined for amiR4416-5p, miR4416-5p STTM transgenic lines and the wild-type in the field at the Purdue Agronomy farm.

Subcellular localization. The GmLe3-encoding vector was transformed into *A. tumefaciens* strain EHA105. A single colony was picked and cultured at 28°C in 3 mL of LB medium supplemented with 50 mg/L rifampicin and 50 mg/L kanamycin until the OD_{600} reached approximately 2.0. The bacterial culture was pelleted, washed with a solution containing 10 mM $MgCl_2$, 10 mM MES (pH5.7), and 200 μM acetosyringone, and incubated in the same solution for an additional 2 h at room temperature. Bacteria containing the mCherry-labeled plasma membrane marker-encoding vector (ABRC stock: CD3-1008) was co-infiltrated with bacteria containing GmLe3-encoding vector. Before infiltration, bacterial cultures were mixed to reach a final OD_{600} of 0.6 for each of the cultures used. The suspension was injected into the abaxial surface of 4–6-week-old *Nicotiana benthamiana* leaves with a needleless syringe. At 72 h after infiltration, the fluorescent signals in detached leaves were imaged using a Zeiss LSM-880 laser-scanning confocal microscope (Zeiss, Thornwood, NY, USA). The excitation wavelength and emission bandwidth recorded for each fluorescent protein were optimized by the default presets in the ZEN 2.6 software (Zeiss) and were as follows: eYFP (excitation 514 nm, emission 519-583 nm), mCherry (excitation 561 nm, emission 580-651 nm).

Root hair curling assay. The roots of V2-stage soybean seedlings were inoculated with *B. diazoefficiens* resuspended in 500 μM NS to an OD_{600} of 0.05. The top 1-cm segment of the primary root of each seedling was cut at 24 hpi and then root hair curling was scored and photographed with a Nikon Eclipse Ti2 microscope (Nikon, NY).

Infection thread quantification. Infection threads were quantified in V1-stage soybean seedlings inoculated with GUS-tagged *B. diazoefficiens* USDA110 at 6 dpi. Specifically, the top five lateral roots were collected and incubated in a staining solution containing 5 mM $K_3Fe(CN)_6$, 5 mM $K_4Fe(CN)_6$, 100 mM sodium phosphate buffer (pH7.0), 1 mM Na_2EDTA (pH8.0), 0.1% (v/v) Triton X-100 and 0.5 mg·mL^{-1} X-Gluc at 37°C for 12 hours. Samples were vacuum-infiltrated for 10 min at the initiation of staining with X-Gluc solution. After staining, the staining solution was removed, and the samples were washed with several changes of 70% (v/v) ethanol until the tissue became clear. Infection threads were counted per centimeter of lateral root length, and images were captured using a Nikon Eclipse Ti2 microscope (Nikon, NY).

Grafting. Grafting was performed as previously described (19) with minor modifications. Seeds of Williams 82 and miR4416-5p STTM were sterilized with chlorine gas overnight and then germinated in sterilized vermiculite. Grafting was initiated 10 d after planting (V0 stage), when the apical meristem of seedlings had reached a height of 12 cm above the vermiculite surface. The hypocotyl was transversely severed with a razor approximately 2 cm below the cotyledon. The lower portion remained in the vermiculite to become the rootstock, and the upper portion became the scion. A vertical incision was made with a razor to a depth of approximately 0.75 cm into the

top center of the rootstock. The scion was razor-trimmed to a V-wedge, which was inserted into the rootstock incision. The graft union was wrapped with Parafilm® M Laboratory Film (Amcor, Zurich, Switzerland). Grafts were placed under greenhouse benches, out of direct sunlight. One week later, successfully grafted plants were inoculated with *B. diazoefficiens* and nodules were counted at 28 dpi. The grafting experiments included four distinct scion/rootstock combinations—Williams 82/miR4416-5p STTM and miR4416-5p STTM/Williams 82 heterografted plants, and Williams 82/Williams 82 and miR4416-5p STTM/miR4416-5p STTM homografted plants. Three biological replicates were conducted, and in each replicate, 12 grafts for each combination were monitored.

GmCEP7 peptide treatment. The conserved CEP domain peptide (“AFRPTTPGNSPGVGH” in *SI Appendix* Fig. S7*A*) with hydroxyprolines (HyP4, HyP7 and HyP11) modifications (hereafter referred to as GmCEP7) was synthesized by Thermo Fisher Scientific Ltd (MA, USA). Sterilized Williams 82 seeds were germinated and grown in sterilized vermiculite pre-moistened with 500 µM NS. To assess the biological activity of the synthetic peptide, a stock solution of GmCEP7 was added to 500 µM NS to a final concentration of 1 µM; deionized water was used as the mock control. V2-stage seedlings were treated in a time-course experiment (Fig. 3 *L* and *M*), and the first trifoliate leaves were harvested at the indicated time points for RNA extraction.

Tracking of synthetic GmCEP7 labeled with stable isotopes. GmCEP7 carrying a stable isotope-labeled $^{13}C_9$, ^{15}N-phenylalanine (hereafter referred to as GmCEP7^{iso}) was synthesized by GenScript USA, Inc (NJ, USA). Sterilized Williams 82 seeds were germinated and grown in sterilized vermiculite moistened with 500 µM NS. To track the movement of the synthetic peptide, a stock solution of GmCEP7^{iso} was added to 500 µM NS to a final concentration of 20 µM and applied to the roots of V2-stage seedlings. The roots and first trifoliate leaves were collected separately at 3 days post-treatment and then ground into a fine powder in liquid nitrogen. The powdered tissues were lysed in GdmCl buffer [6 M guanidinium chloride, 100 mM Tris-HCl (pH 8.5)] at a ratio of 100 mg tissue to 400 µL buffer. Samples were incubated on ice for 30 min, sonicated at 15% amplitude for 3 × 30 s with 1-min intervals, and centrifuged at 15,000 rpm for 15 min at 4°C. The supernatant was collected and diluted tenfold with Milli-Q water to reduce the GdmCl concentration. Trifluoroacetic acid (TFA) was added to a final concentration of 1%, and the samples were centrifuged at 10,000 × g for 20 min at 4°C to precipitate proteins. The peptide-containing supernatant was dried by vacuum centrifugation and desalted using custom-packed solid-phase extraction tips with C18 silica gel resin (Sorbtech, 60 Å pore size, 40–63 µm particle size).

For HPLC fractionation, 500 µg of desalted peptides were separated using an Agilent HPLC system (1200/1260 Infinity) equipped with a Waters XBridge™ BEH130 C18 column (4.6 × 150 mm, 3.5 µm particle size). Peptides were separated at a flow rate of 0.3 mL/min using solvent A (0.1% formic acid in water) and solvent B (0.1% formic acid in 100% acetonitrile). An isocratic flow of 100% solvent A was applied from 0 to 2 min. From 2 to 12 min, a linear gradient to 10% solvent B was applied, followed by a gradient to 20% B from 12 to 22 min, to 30% B from 22 to 32 min, and to 80% B from 32 to 35 min. The gradient was held at 100% solvent B from 36 to 50 min, followed by re-equilibration with 100% solvent A until 51 min. Fractions were collected and dried by vacuum centrifugation prior to LC-MS/MS analysis.

Dried peptides were dissolved in 0.1% formic acid and loaded onto Evotips Pure (Evosep) according to the manufacturer's instructions. Samples were analyzed using a timsTOF HT mass spectrometer (Bruker Daltonics) coupled to an Evosep One LC system (Evosep). A reversed-phase C18 column (8 cm × 150 µm ID, 1.5 µm particle size; Evotip Pure Columns) was connected to a ZDV Sprayer 20 µm emitter (Bruker) within a Captive Spray source (Bruker) operated at 60°C. The mobile phases consisted of 0.1% formic acid in LC-MS grade water (solvent A) and 0.1% formic acid in LC-MS grade acetonitrile (solvent B). Samples were analyzed using a 60 SPD gradient (21-min gradient time, 1 µL/min flow rate). The accumulation and ramp times for the dual TIMS analyzer were set to 100 ms as a duty cycle. Full MS data were acquired in a mass range of 100–1700 m/z and mobility range of 0.6–1.7 Vs/cm². The collision energy was ramped as a

function of increasing mobility from 20 eV at 0.6 Vs/cm² to 75 eV at 1.6 Vs/cm². For targeted detection of GmCEP7^{iso} peptide, parallel reaction monitoring (PRM-PASEF) was performed. Transition lists were generated from DDA analysis of the synthetic GmCEP7^{iso} peptide and included retention time, precursor m/z, charge state, and ion mobility values.

Syntenic analysis of the *MIR4416* loci. To investigate syntenic relationships surrounding the *MIR4416* locus, reference genomes for *Arabidopsis thaliana* (Araport 11) (20), *Medicago truncatula* (Mt4.0v1) (21), *Lotus japonicus* (Lj1.0v1) (22), *Phaseolus vulgaris* (v2.1, available at http://phytozome.jgi.doe.gov/), *Cajanus cajan* (23) and *Glycine max* (v2.1) (24) were retrieved. BLASTP (v.2.2.30+) (25) was applied to calculate pairwise similarities (e-value $< 1 \times 10^{-5}$) for genes among different species. Then, collinearity between each pair of species was detected by WGDI (26). Syntenic genes around the *MIR4416* loci were extracted from the collinearity file. The microsynteny plot among the species was then generated by utilizing JCVI (27).

Quantification and statistical analysis. Please refer to the figure legends for descriptions of sample sizes and statistical tests performed. Differences were considered statistically significant when the *p*-value was less than 0.05. Illustrations were prepared using Adobe Illustrator.

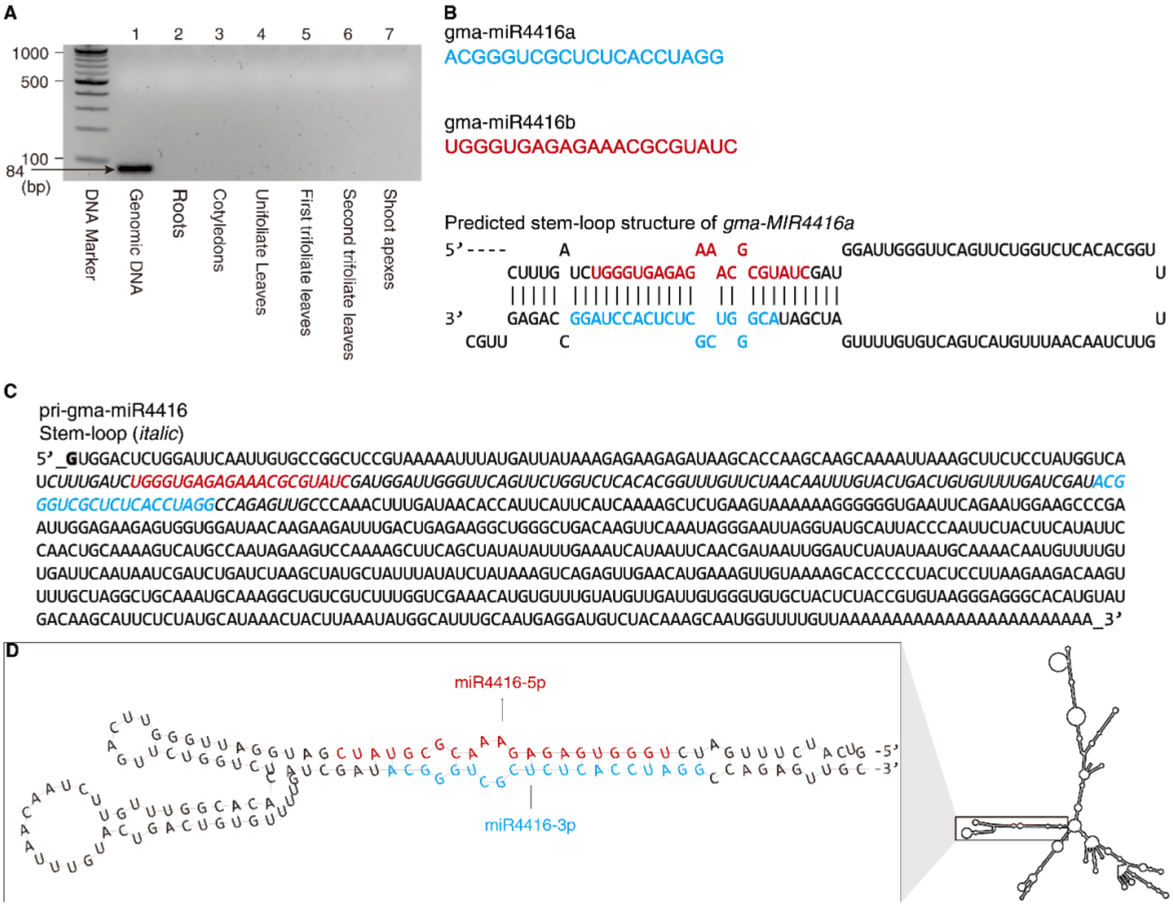

Figure S1. Sequence, expression, and structure of *MIR4416* transcripts. (*A*) Expression of *gma-MIR4416b* in different V2-stage soybean tissues. PCR products were loaded on 2% agarose gel containing SYBR^{TM} Safe DNA Gel Stain and visualized under blue light. Genomic DNA was used as a positive control. (*B*) Predicted stem-loop structure of *gma-MIR4416a* based on miRBase. The *gma-MIR4416a* contains a single copy of gma-miR4416a (blue) and gma-miR4416b (red). (*C*) Primary transcript of gma-miR4416 (pri-gma-miR4416) was confirmed experimentally by 5’ and 3’ RACE. The transcription start site is shown in bold, the stem-loop structure is in italics, and gma-miR4416a and gma-miR4416b are shown in blue and red, respectively. (*D*) Predicted secondary structure of pri-gma-miR4416 and the resulting miR4416-5p (red) and miR4416-3p (blue).

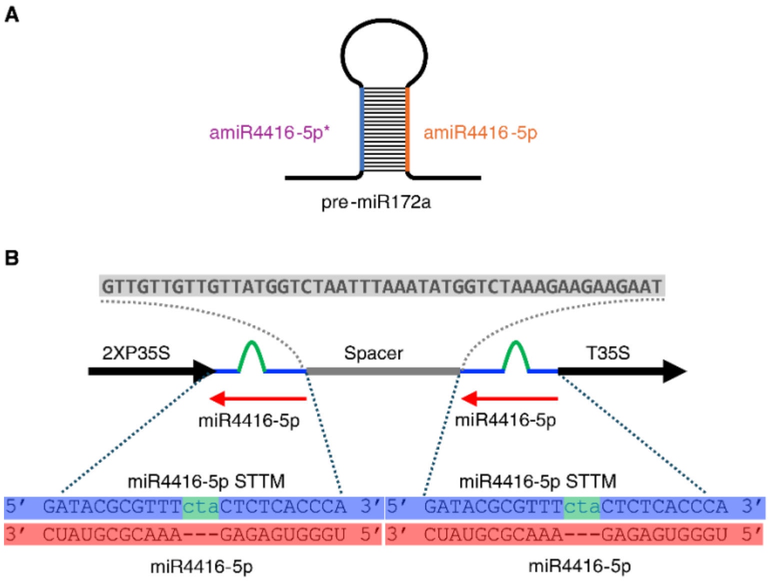

Figure S2. Diagrammatic illustration of miR4416-5p amiR and STTM structures. (*A*) The artificial *MIRNA* construct was generated from soybean *MIR172a* by replacing miR172a/miR172a* with amiR4416-5p/amiR4416-5p*, which are identical or complementary to miR4416-5p. (*B*) Structure of miR4416-5p STTM. Green indicates the bulge sequences in the STTM that are not complementary to miR4416-5p.

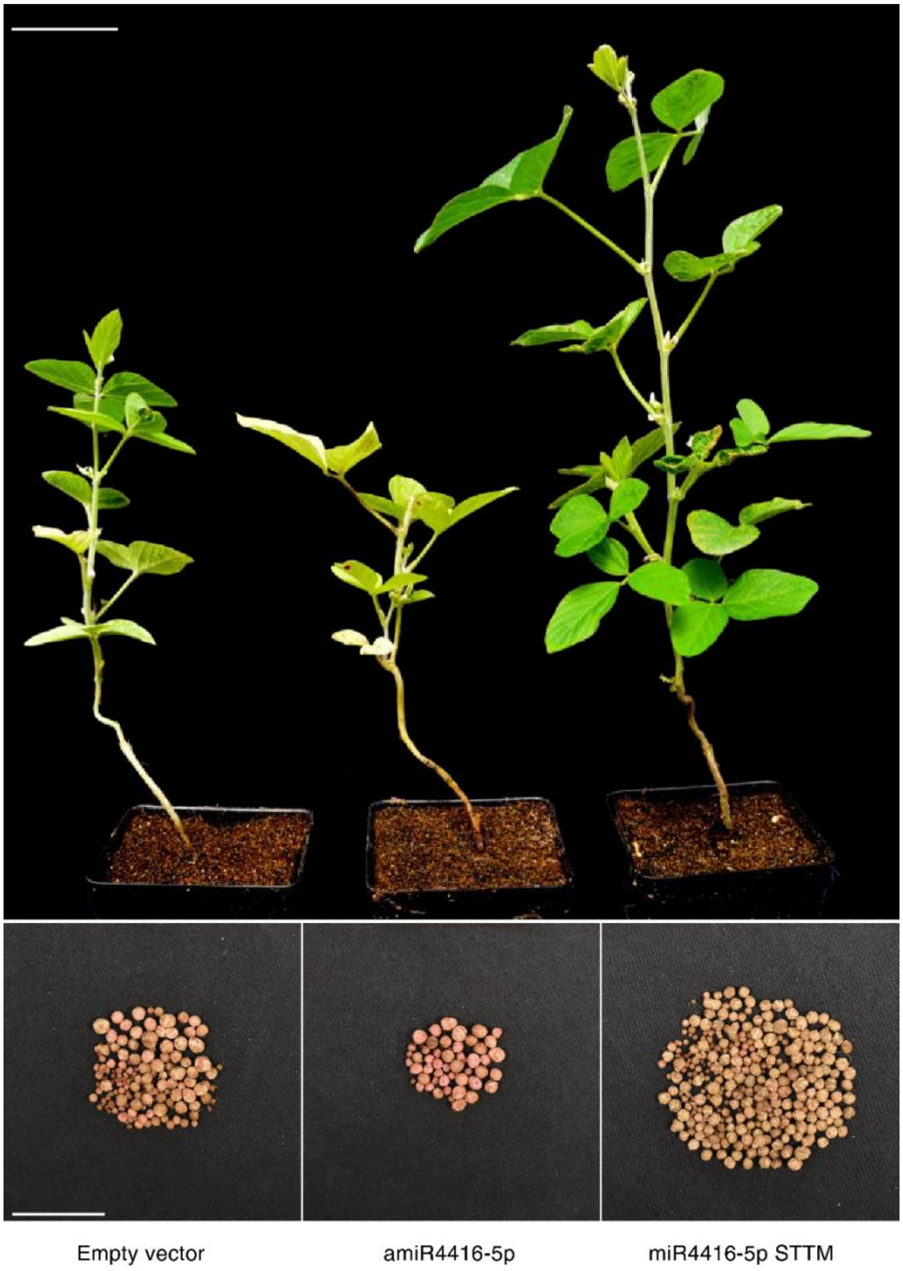

Figure S3. Above-ground growth and nodulation in control, amiR4416-5p and miR4416-5p STTM composite plants. Hairy roots induced by *Agrobacterium rhizogenes* (transformed with empty vector) were used as controls. Plants were photographed 28 dpi with *B. diazoefficiens* and nodules were then harvested. Scale bars: 5 cm (top), 2 cm (bottom).

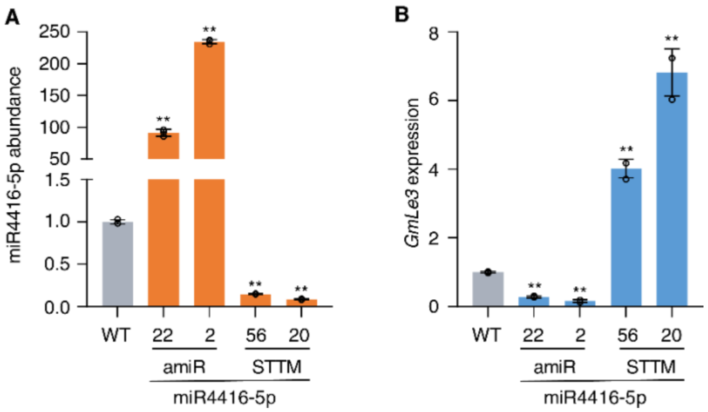

Figure S4. miR4416-5p abundance and *GmLe3* expression in root hairs of stable miR4416-5p transgenic lines. (*A*) The relative abundance of miR4416-5p in root hairs of stable miR4416-5p transgenic plants (n = 3). Statistical differences were determined with Student's t test. p values: $^{**}p \leq 0.01$. The data are reported as the mean ± s.d. (*B*) The expression levels of *GmLe3* in root hairs of stable miR4416-5p transgenic plants (n = 3). Statistical differences were determined with Student's t test. The data are reported as the mean ± s.d.

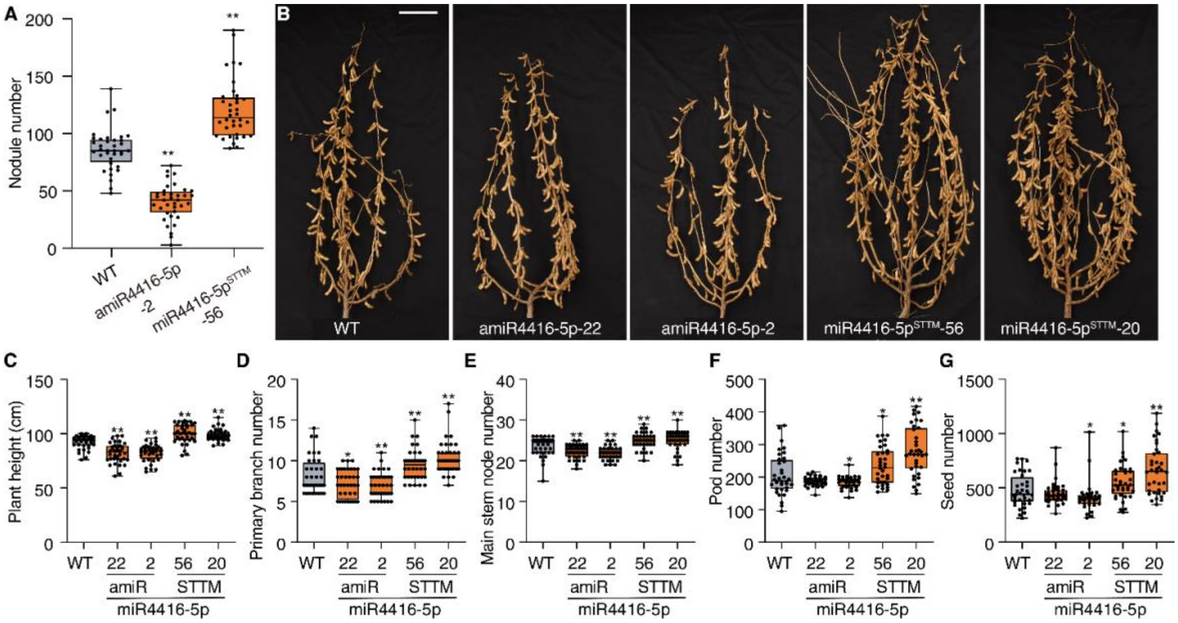

Figure S5. Assessment of the effects of miR4416-5p on agronomic traits. (*A*) Changes in nodule numbers (28 dpi) of stable amiR4416-5p and miR4416-5p^{STTM} plants compared with the WT. For all box-and-whisker plots presented in this study, the lines represent the median, the dots represent the data points ($n = 36$), the edges of the boxes define the interquartile ranges, and the whiskers represent the minimum and maximum values. Statistical differences were determined with Student's *t* test. *p* values: $^{**}p \leq 0.01$. The experiment was repeated three times independently. (*B*) Gross morphologies of representative WT soybean, amiR4416-5p and miR4416-5p^{STTM} stable transgenic lines. Scale bar, 10 cm. (*C*-*G*) Changes in plant height (*C*), primary branch number (*D*), main stem node number (*E*), pod number (*F*), and seed number (*G*) of amiR4416-5p and miR4416-5p^{STTM} stable transgenic plants compared with the WT. For all box-and-whisker plots presented in this study, the lines represent the median, the dots represent the data points ($n = 36$), the edges of the boxes define the interquartile ranges, and the whiskers represent the minimum and maximum values. Statistical differences were determined with Student's *t* test. *p* values: $^{*}p \leq 0.05$; $^{**}p \leq 0.01$.

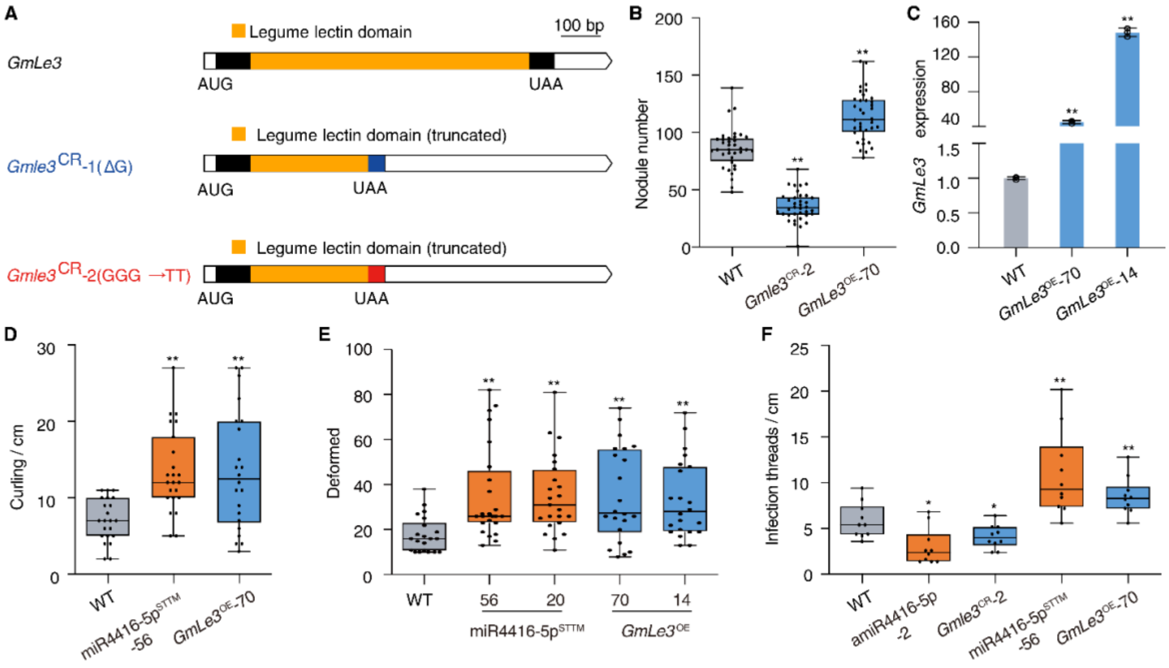

Figure S6. Assessment of the effects of miR4416-5p-*GmLe3* module on rhizobial infection and nodulation. (*A*) Genome editing caused mutations in the *GmLe3* coding region and resulted in premature termination of the encoded proteins. Different transcript forms were confirmed experimentally by RT-PCR followed by Sanger sequencing. (*B*) Changes in nodule numbers (28 dpi) of stable *Gmle3* mutants and *GmLe3*-overexpression (*GmLe3*^{OE}) plants compared with the WT. For all box-and-whisker plots presented in this study, the lines represent the median, the dots represent the data points ($n = 36$), the edges of the boxes define the interquartile ranges and the whiskers represent the minimum and maximum values. Statistical differences were determined with Student's *t* test. *p* values: $^{**}p \leq 0.01$. The experiment was repeated three times independently. (*C*) The expression levels of *GmLe3* in root hairs of stable *GmLe3*-overexpression transgenic plants ($n = 3$). Statistical differences were determined with Student's *t* test. The data are reported as the mean ± s.d. (*D*) Changes in the numbers of curled root hairs (24 hpi) in stable miR4416-5p^{STTM} and *GmLe3*^{OE} plants compared with the WT. WT, $n = 22$; miR4416-5p^{STTM}-56, $n = 23$; *GmLe3*^{OE}-70, $n = 22$. Statistical differences were determined with Student's *t* test. *p* values: $^{**}p \leq 0.01$. The experiment was repeated three times independently. (*E*) Changes in deformed root hairs of stable miR4416-5p^{STTM} and *GmLe3*^{OE} plants compared with the WT. WT, $n = 21$; miR4416-5p^{STTM}-56, $n = 24$; miR4416-5p^{STTM}-20, $n = 23$; *GmLe3*^{OE}-70, $n = 22$; *GmLe3*^{OE}-14, $n = 22$. Statistical differences were determined with Student's *t* test. *p* values: $^{**}p \leq 0.01$. The experiment was repeated three times independently. (*F*) Changes in the numbers of infection threads in stable amiR4416-5p, *Gmle3* mutants, miR4416-5p^{STTM} and *GmLe3*^{OE} plants compared with WT. $n = 10$. Statistical differences were determined with Student's *t* test. *p* values: $^{*}p \leq 0.05$; $^{**}p \leq 0.01$. The experiment was repeated three times independently.

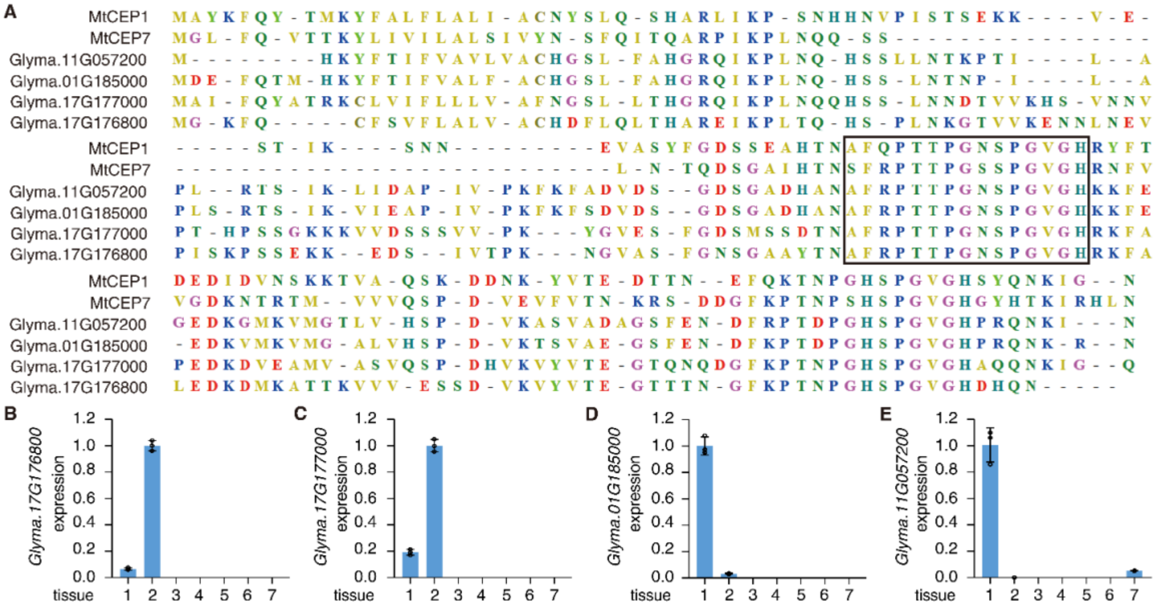

Figure S7. Characterization of GmCEP7 and its precursor genes. (*A*) Multiple sequence alignment (MSA) of the amino acid sequences for MtCEP1, MtCEP7, and their corresponding orthologs in soybean. The conserved 15-aa CEP domains are highlighted with a black box. (*B*-*E*) Expression of *Glyma.17G176800* (*B*), *Glyma.17G177000* (*C*), *Glyma.01G185000* (*D*), and *Glyma.11G057200* (*E*) in different tissues of the wild type (WT) plants (n = 3). Tissue samples were collected from uninfected V2-stage soybean seedlings. Roots (1), stems (2), cotyledons (3), unifoliate leaves (4), first trifoliate leaves (5), second trifoliate leaves (6), and shoot apexes (7). The data are reported as the mean ± s.d.

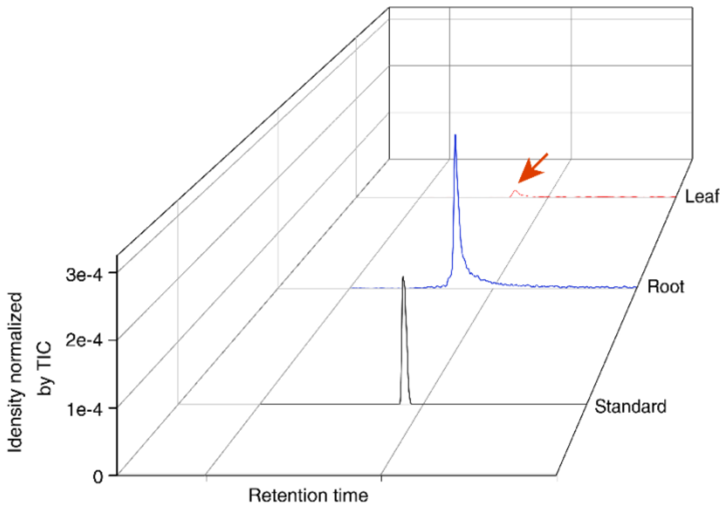

Figure S8. Detection of stable isotope-labeled synthetic GmCEP7 peptide (GmCEP7^{iso}) by LC-MS/MS. Extracted ion chromatograms showing the retention profile of the labeled peptide (*m/z* 518.2571) in peptide standard stock, root extracts and leaf extracts from soybean plants treated with 20 μM GmCEP7^{iso}.

Supplementary Tables

Table S1. Expression levels (CPM) of the gma-miR4416b and its predicted target gene *Glyma.02G156800* in uninoculated roots, 10 dpi and 20 dpi nodules.

miRNA/Gene	miRNA abundance/transcript levels revealed by sRNA-seq/RNA-seq*		
	Uninoculated roots	Nodules (10 dpi)	Nodules (20 dpi)
gma-miR4416b	7.5	1.3	2.0
Glyma.02G156800	4.0	71.8	80.3

*Detected microRNA copy numbers or transcript levels per million reads.

Table S2. Primers used in this study.

**Primer name**	**Sequence (5'-3')**	**Purpose**
Universal Reverse primer	GTGCAGGGTCCGAGGT	Stem-loop RT-qRT-PCR
miR4416-5p RT primer	GTCGTATCCAGTGCAGGGTCCGAGGTATTCGCACTGGATACGACgatacg	Stem-loop RT-qRT-PCR
miR4416-5p F primer	cgcgtgTGGGTGAGAGAAACG	Stem-loop RT-qRT-PCR
miR172c RT primer	GTCGTATCCAGTGCAGGGTCCGAGGTATTCGCACTGGATACGACctgcag	Stem-loop RT-qRT-PCR
miR172c F primer	tcgcgtgGGAATCTTGATGATG	Stem-loop RT-qRT-PCR
GmELF1b qRT F	GTTGAAAAGCCAGGGGACA	qRT-PCR
GmELF1b qRT R	TCTTACCCCTTGAGCGTGG	qRT-PCR
GmLe3 qRT F	TCCGTTGAAACCCATGATGTGA	qRT-PCR
GmLe3 qRT R	CATTGAGCACAAAGCTTGGAAGA	qRT-PCR
PrecursorCh 03 qRT F	CAGGGCTTACTAGTTCACACAGT	qRT-PCR
PrecursorCh 03 qRT R	GACCCGTATCGATCAAAACACAC	qRT-PCR
MIR4416 qRT F	ACATGAAAGTTGTAAAAGCACCCC	qRT-PCR
MIR4416 qRT R	TTGCAGCCTAGCAAAACTTGTC	qRT-PCR
Glyma.01G185000 qRT F	CTTCCTTGGCATGTCTTTACGTG	qRT-PCR
Glyma.01G185000 qRT R	GGTCTGGTGCAGAACAAAAGAAA	qRT-PCR
Glyma.11G057200 qRT F	CTTTTTGCTCATGGAAGGCAGAT	qRT-PCR
Glyma.11G057200 qRT R	TGAATTTTGGGACAATTGGTGCG	qRT-PCR
Glyma.17G176800 qRT F	GGTCACCGAAAATTTGCACTAGAA	qRT-PCR
Glyma.17G176800 qRT R	GTCGTTGTCCCCTCAGTAACATA	qRT-PCR
Glyma.17G177000 qRT F	ACTCATCATCCGTGGTACCAAAA	qRT-PCR
Glyma.17G177000 qRT R	GTCTGAAAGCATTTGTGTCCGAA	qRT-PCR
miR4416-5p STTM Forward1	GATACGCGTTTctaCTCTCACCCAGTTGTTGTTGTTATGGTCTAATTTAAATATGGTCTAAAGAAGAAGAATGATACGCGTTTctaCTCTCACCCA	STTM construction for hairy root induction
miR4416-5p STTM Reverse1	ctagTGGGTGAGAGtagAAACGCGTATCATTCTTCTTCTTTAGACCATATTTAAATTAGACCATAACAACAACAACTGGGTGAGAGtagAAACGCGTATCagct	STTM construction for hairy root induction
amiR4416-5p Forward1	TTAACAGTCGTTATTTGCGGTGGGTGAGAGAAACGCGTATCCATGCAAGCGCAGGTGGTGGGTGGGACTTGATGCAATCTAAGTGCTGTGCCAGCCAAGCCATAGGTCTTTTGGAACTGGATACGCGTTTCTCTCACCCACAGCCATAAACGACTTCAC	Artificial microRNA construction for hairy root induction
amiR4416-5p Reverse1	ctagGTGAAGTCGTTTATGGCTGTGGGTGAGAGAAACGCGTATCCAGTTCCAAAAGACCTATGGCTTGGCTGGCACAGCACTTAGATTGCATCAAGTCCCACCCACCACCTGCGCTTGCATGGATACGCGTTTCTCTCACCCACCGCAAATAACGACTGTTAAagct	Artificial microRNA construction for hairy root induction

miR4416-5p STTM Forward2	ccggGATACGCGTTTctaCTCTCACCCAGTTGTTGTTGTTATGGTCTAATTTAAATATGGTCTAAAGAAGAAGAATGATACGCGTTTctaCTCTCACCCA	STTM construction for stable transformation
miR4416-5p STTM Reverse2	gatcTGGGTGAGAGtagAAACGCGTATCATTCTTCTTCTTTAGACCATATTTAAATTAGACCATAACAACAACAACTGGGTGAGAGtagAAACGCGTATC	STTM construction for stable transformation
amiR4416-5p Forward2	ccggTTAACAGTCGTTATTTGCGGTGGGTGAGAGAAACGCGTATCCATGCAAGCGCAGGTGGTGGGTGGGACTTGATGCAATCTAAGTGCTGTGCCAGCCAAGCCATAGGTCTTTTGGAACTGGATACGCGTTTCTCTCACCCACAGCCATAAACGACTTCAC	Artificial microRNA construction for stable transformation
amiR4416-5p Reverse2	gatcGTGAAGTCGTTTATGGCTGTGGGTGAGAGAAACGCGTATCCAGTTCCAAAAGACCTATGGCTTGGCTGGCACAGCACTTAGATTGCATCAAGTCCCACCCACCACCTGCGCTTGCATGGATACGCGTTTCTCTCACCCACCGCAAATAACGACTGTTAA	Artificial microRNA construction for stable transformation
GmLe3 Reverse GSP1	CCGGTGGTGGCAGAGAACCCAACGC	5' RACE
GmLe3 Reverse GSP2	ACCCCCACGATGCCGTTTTGACGGAC	5' RACE
GmLe3 Forward GSP1	CAAAGTTGGCAGCAACGGCGTGCCC	3' RACE
GmLe3 Forward GSP2	TCGGTCGTGCCCTTTACGCTGCCCC	3' RACE
PrecursorCh 19 Reverse GSP1	CCACCACTCTTCTCCAATTCGGGCT	5' RACE
PrecursorCh 19 Reverse GSP2	TCTGGCCTAGGTGAGAGCGACCCGT	5' RACE
PrecursorCh 19 Forward GSP1	ACGCGTATCGATGGATTGGGTTCAGT	3' RACE
PrecursorCh 19 Forward GSP2	TGGGTTCAGTTCTGGTCTCACACGGT	3' RACE
*GmLe3*_pGES201_F	ggattgTTTAAAGGATGTAGCCCAGC	CRISPR/Cas9 construction
*GmLe3*_pGES201_R	aaacGCTGGGCTACATCCTTTAAAca	CRISPR/Cas9 construction
*GmLe3*_genotyping_F	TACACAACCCGATGAAAGTCCTA	Mutation site (introduced by genome-editing) detection
*GmLe3*_genotyping_R	TATAGGTAACGAGAATCTCCGCC	Mutation site (introduced by genome-editing) detection
*GmLe3*_CDS_F	caccatttacgaacgatagcATGGCCACCTCCAACTTCTC	Overexpression construction
*GmLe3*_CDS_R	actggtgatttttgcggactTTAGATGGCCTCATTGAGCAC	Overexpression construction
GmLe3 N SL F	ttctgcccaaattcgcgcATGGCCACCTCCAACTTC	Subcellular localization
GmLe3 N SL R	ctcctcgcccttgctcactccacctcctccaccacctccGATGGCCTCATTGAGCAC	Subcellular localization
GmCEP7 RNAi F1	tttacaattaccatggggTAAGCAGAACCAGCATTAAAG	RNA interference
GmCEP7 RNAi R1	catttaaatcatcgattgggTCTTGTTTTGACGAGGATG	RNA interference
GmCEP7 RNAi F2	tttggatcctaggtgagtTCTTGTTTTGACGAGGATG	RNA interference
GmCEP7 RNAi R2	cgggtcttaattaactctTAAGCAGAACCAGCATTAAAG	RNA interference

*Glyma.11G057200*_CDS_F	gcgcGGCGCGCCatgcacaaatattttactatcttcgtc	Overexpression construction
*Glyma.11G057200*_CDS_R	gcgcTCTAGAttaattaatcttgttttgacgaggatg	Overexpression construction

SI References

1. R. L. Bernard, C. R. Cremeens, Registration of ‘Williams 82’ Soybean. *Crop Sci.* **28**, cropsci1988.0011183X002800060049x (1988).
2. R. Di, V. Purcell, G. B. Collins, S. A. Ghabrial, Production of transgenic soybean lines expressing the bean pod mottle virus coat protein precursor gene. *Plant Cell Rep.* **15**, 746-750 (1996).
3. P. van Berkum, Evidence for a third uptake hydrogenase phenotype among the soybean Bradyrhizobia. *Appl. Environ. Microbiol.* **56**, 3835-3841 (1990).
4. M. Libault *et al.*, Complete transcriptome of the soybean root hair cell, a single-cell model, and its alteration in response to *Bradyrhizobium japonicum* infection. *Plant Physiol.* **152**, 541-552 (2009).
5. P. Huang *et al.*, An efficient *Agrobacterium rhizogenes*-mediated hairy root transformation method in a soybean root biology study. *Int. J. Mol. Sci.* **23**, 12261 (2022).
6. Y. He, T. Zhang, H. Sun, H. Zhan, Y. Zhao, A reporter for noninvasively monitoring gene expression and plant transformation. *Hortic. Res.* **7**, 152 (2020).
7. S. Li *et al.*, Unidirectional movement of small RNAs from shoots to roots in interspecific heterografts. *Nat. Plants* **7**, 50-59 (2021).
8. B. Ren, X. Wang, J. Duan, J. Ma, Rhizobial tRNA-derived small RNAs are signal molecules regulating plant nodulation. *Science* **365**, 919-922 (2019).
9. B. Langmead, C. Trapnell, M. Pop, S. L. Salzberg, Ultrafast and memory-efficient alignment of short DNA sequences to the human genome. *Genome Biol.* **10**, R25 (2009).
10. D. Zhang *et al.*, Elevation of soybean seed oil content through selection for seed coat shininess. *Nat. Plants* **4**, 30-35 (2018).
11. E. Varkonyi-Gasic, R. Wu, M. Wood, E. F. Walton, R. P. Hellens, Protocol: a highly sensitive RT-PCR method for detection and quantification of microRNAs. *Plant Methods* **3**, 12 (2007).
12. X. Dai, P. X. Zhao, psRNATarget: a plant small RNA target analysis server. *Nucleic Acids Res.* **39**, W155-W159 (2011).
13. J. Ping *et al.*, *Dt2* Is a gain-of-function MADS-domain factor gene that specifies semideterminacy in soybean. *Plant Cell* **26**, 2831-2842 (2014).
14. J. Yan *et al.*, Effective small RNA destruction by the expression of a short tandem target mimic in *Arabidopsis*. *Plant Cell* **24**, 415-427 (2012).
15. Q.-W. Niu *et al.*, Expression of artificial microRNAs in transgenic *Arabidopsis thaliana* confers virus resistance. *Nat. Biotechnol.* **24**, 1420-1428 (2006).
16. M. Bai *et al.*, Generation of a multiplex mutagenesis population via pooled CRISPR-Cas9 in soya bean. *Plant Biotechnol. J.* **18**, 721-731 (2020).
17. Y. Naito, K. Hino, H. Bono, K. Ui-Tei, CRISPRdirect: software for designing CRISPR/Cas guide RNA with reduced off-target sites. *Bioinformatics* **31**, 1120-1123 (2014).
18. Z. Y. Song *et al.*, Screening Chinese soybean genotypes for *Agrobacterium*-mediated genetic transformation suitability. *J Zhejiang Univ Sci B* **14**, 289-298 (2013).
19. V. R. Pantalone, G. J. Rebetzke, J. W. Burton, T. E. Carter, D. W. Israel, Soybean PI 416937 root system contributes to biomass accumulation in reciprocal grafts. *Agron. J.* **91**, 840-844 (1999).
20. C.-Y. Cheng *et al.*, Araport11: a complete reannotation of the *Arabidopsis thaliana* reference genome. *Plant J.* **89**, 789-804 (2017).
21. H. Tang *et al.*, An improved genome release (version Mt4.0) for the model legume *Medicago truncatula*. *BMC Genomics* **15**, 312 (2014).
22. H. Li, F. Jiang, P. Wu, K. Wang, Y. Cao, A high-quality genome sequence of model legume *Lotus japonicus* (MG-20) provides insights into the evolution of root nodule symbiosis. *Genes* (Basel) **11**, 483 (2020).
23. R. K. Varshney *et al.*, Draft genome sequence of pigeonpea (*Cajanus cajan*), an orphan legume crop of resource-poor farmers. *Nat. Biotechnol.* **30**, 83-89 (2012).
24. J. Schmutz *et al.*, Genome sequence of the palaeopolyploid soybean. *Nature* **463**, 178-183 (2010).

25. S. F. Altschul, W. Gish, W. Miller, E. W. Myers, D. J. Lipman, Basic local alignment search tool. *J. Mol. Biol.* **215**, 403-410 (1990).
26. P. Sun *et al.*, WGDI: A user-friendly toolkit for evolutionary analyses of whole-genome duplications and ancestral karyotypes. *Mol. Plant* **15**, 1841-1851 (2022).
27. H. Tang *et al.*, JCVI: A versatile toolkit for comparative genomics analysis. *iMeta* **3**, e211 (2024).
